# Development of a rapid scabies immunodiagnostic assay based on transcriptomic analysis of *Sarcoptes scabiei* var. *nyctereutis*

**DOI:** 10.1038/s41598-021-85290-7

**Published:** 2021-03-19

**Authors:** Teruo Akuta, Daisuke Minegishi, Nobuhide Kido, Keitaro Imaizumi, Shinji Nakaoka, Shin-Ichiro Tachibana, Kenji Hikosaka, Fumi Hori, Masataka, Nakagawa, Chiaki Sakuma, Yuki Oouchi, Yu Nakajima, Sohei Tanaka, Tomoko Omiya, Kouki Morikaku, Minori Kawahara, Yoshifumi Tada, Hiroshi Tarui, Takafumi Ueda, Takane Kikuchi-Ueda, Yasuo Ono

**Affiliations:** 1 Research Division, Kyokuto Pharmaceutical Industrial Co., Ltd., 3333-26, Aza-Asayama, Kamitezuna, Takahagi-shi, Ibaraki 318-0004 Japan; 2 Department of Microbiology and Immunology, Teikyo University School of Medicine, 2-11-1 Kaga, Itabashi-ku, Tokyo 173-8605 Japan; 3 Research Institute of Bio-System Informatics, Tohoku Chemical Co., Ltd, 6-15-5, Mitake, Morioka, Iwate 020-0122 Japan; 4 Kanazawa Zoological Gardens, 5-15-1 Kamariyahigashi, Kanazawa-ku, Yokohama, Kanazawa 236-0042 Japan; 5 Faculty of Advanced Life Science, Hokkaido University, Kita 10, Nishi 8, Kita-ku, Sapporo, 060-0810 Japan; 6 Genome Information Research Center, Research Institute for Microbial Diseases, Osaka University, 3-1 Yamadaoka, Suita, Osaka 565-0871 Japan; 7 Department of Infection and Host Defense, Graduate School of Medicine, Chiba University, 1-8-1 Inohana, Chuo-ku, Chiba, 260-8670 Japan; 8 Division of Genomic Technologies, Center for Life Science Technologies, RIKEN Yokohama Institute, 1-7-22 Suehiro-cho, Yokohama, 230-0045 Japan

**Keywords:** Biochemistry, Biological techniques, Biotechnology, Evolution, Molecular biology, Biomarkers, Diseases, Medical research, Molecular medicine

## Abstract

Scabies is a highly contagious skin disease caused by the mite *Sarcoptes scabiei* that affects many mammals. However, the sensitivity of traditional tests for scabies diagnosis in humans is less than 50%. To simplify the diagnosis of scabies, methods that are simple, sensitive, specific, and cost-effective are required. We developed an immunodiagnostic test based on *S. scabiei* var. *nyctereutis* RNA-seq data collected from Japanese raccoon dogs with sarcoptic mange. Three candidate antigens—a highly expressed hypothetical protein “QR98_0091190,” another mite allergen known as “SMIPP-Cc,” and an abundant “vitellogenin-like protein”—were evaluated by western-blot analysis. A lateral flow immunoassay, using specific antibodies against the vitellogenin-like protein, successfully detected scabies in the skin flakes of *S. scabiei*-infected raccoon dogs. This assay can potentially diagnose scabies more accurately in wildlife, as well as in humans.

## Introduction

Scabies is a highly contagious skin disease caused by the itch mite *Sarcoptes scabiei* (*Acari*: *Sarcoptidae*) that affects over 100 species of mammals, including humans^1,2^. Additionally, *S. scabiei* negatively affects human public–health and global economic losses related to animal production^3,4^. In developed countries, scabies outbreaks are common in residential areas, as well as nursing homes, where they cause significant morbidity and distress. Moreover, there is widespread misdiagnosis of scabies and the management of outbreaks is costly. Globally, more than 200 million people are affected by this disease, with a particularly high prevalence observed in resource-poor tropical regions. Scabies was added to the WHO Neglected Tropical Diseases portfolio in 2017, and the 2020 IACS (The International Alliance for the Control of Scabies) Consensus Criteria for the Diagnosis of Scabies can be implemented in scabies research and mapping projects^5^. As mentioned above, despite the international importance of this infectious disease, adequate diagnostic methods have not been established to date.

The diagnostic sensitivity of traditional methods involving the microscopic examination of skin scrapings was reported to be less than 50%^6,7^. Scabies infestation is mostly diagnosed within a few days based on clinical signs and re-testing. The detection of visible lesions using dermoscopy or microscopy can be challenging, as these lesions are often obscured by eczema or impetigo or are atypical. Several diagnostic techniques that do not require visualization of the mite are under investigation in animals and humans^6,7^. Examples of these methods include polymerase chain reaction (PCR), real time PCR, ELISA (serodiagnostics), and immunohistochemistry, among others^6,8–15^. Well-designed and properly conducted studies are necessary to determine the accuracy and utility of these methods. PCR is considerably specific and sensitive; however, it is disadvantageous owing to the requirement for specialized equipment, and its unsuitability for the detection of genes in cases where the mite DNA is not present in the infected area of the skin. While ELISA is also highly sensitive and accurate, currently, there are only reports of serodiagnostic methods. ELISA is disadvantageous as it is cumbersome, time-consuming, and requires a microplate reader.

In this study, we aimed to develop a simple, rapid, sensitive, specific, and cost-effective method for the diagnosis of scabies. Immunochromatography for scabies was completed after a four-step study, involving: (1) transcriptional analysis of the mange mites, (2) selection of the mite antigens using our newly obtained data in conjunction with previously published genomic and proteomic data^16–18^, (3) preparation and evaluation of antibodies specific to the selected antigens, and (4) construction of a lateral flow device for the evaluation of the clinical sample.

We used *Sarcoptic scabiei* var. *nyctereutis* collected from Japanese raccoon dogs; these dogs are severely affected by scabies, making the collection of mites relatively easy^19^. In a previous study, it was indicated that the mitochondrial DNA sequence of *S. scabiei* var. *nyctereutis* shared 99% identity with the mitochondrial genome sequences of *S. scabiei* var. *canis*, var. *hominis*, and var. *suis*^20^. Findings from *S. scabiei* var. *nyctereutis*, could, therefore, be extrapolated to *S. scabiei* var. *hominis* as well as other animal varieties and could be easily extrapolated to the other *S. scabiei* variants. In this study, we performed the transcriptional analysis of *S. scabiei* var. *nyctereutis*, identified candidate antigens for the development of an immunoassay, and prepared specific antibodies against the three candidate antigens (QR98_0091190, SMIPP-Cc, vitellogenin-like protein). We particularly focused on vitellogenin-like protein. Vitellogenin-like protein is a soluble protein that is expressed at high levels in mites^18^. The protein is considered to be processed from a single large (200–700 kDa) precursor phosphoglycolipoprotein that form oligomers of one to four subunits consisting of large (140–190 kDa) and small (40–60 kDa) subunits,, which have been shown to remain stable (based on evidence from insect studies)^21^. Finally, we evaluated the utility of our lateral immunoassay using the skin scrapings of some scabies-infected raccoon dogs.

## Results

### Concept of this study

**Figure 1:**
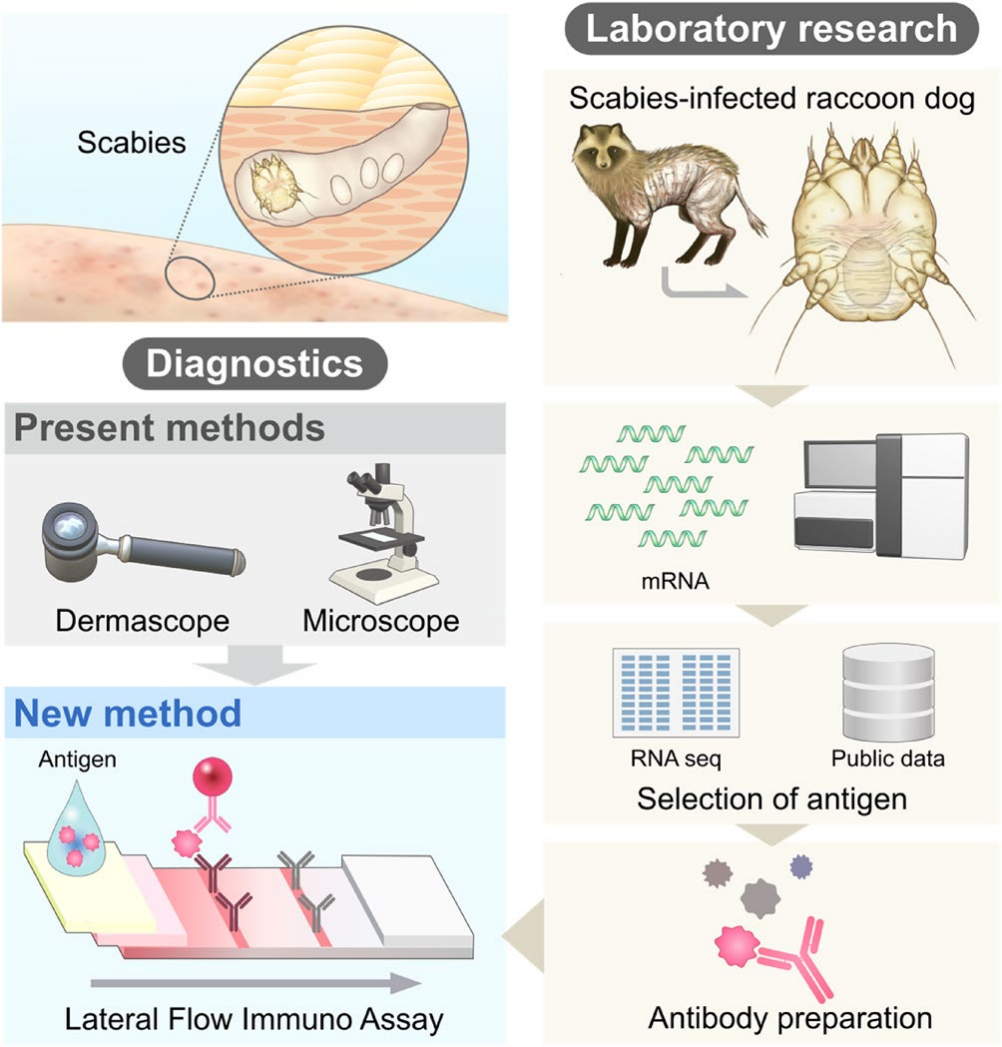
Schematic overview of this study for the development of a lateral immunoassay for scabies diagnosis. This is an original diagram drawn by co-author Chiaki Sakuma and us.

The detection rates for scabies infestation under skin using present diagnostic methods employing dermoscopy and microscopy are commonly less than 50%^6,7^, making it difficult to diagnose the disease accurately (Fig. 1). Therefore, we conducted this study to develop a lateral flow immunoassay with a newly prepared antibody against candidate antigens identified using the RNA-seq data of *S. scabiei* var. *nyctereutis* collected from Japanese raccoon dogs with severe mange.

### Sequencing and assembly

In total, 162,918 contigs, with a maximum length of 30,727 bp and a minimum length of 201 bp (N50 = 4,964 bp) were generated by de novo assembly (Table 1). Among these, 98.4% (109,185/110,911) were homologous to *S. scabiei* var. *canis* gene sequences and 110,033 were annotated. Data of raw sequence reads and assembled contigs obtained in this study were deposited into GenBank/EMBL/DDBJ, with accession numbers PSUB008186 and IACW01000001-IACW01044773, respectively.

**Table 1:** Statistics of the transcriptomic analysis for *Sarcoptes scabiei* var. *nyctereutis* collected from wild Japanese raccoon dogs.

Description of transcripts	Value
Number of transcripts	162,918
Maximum transcript length (bp)	30,727
Minimum transcript length (bp)	201
Mean transcript length (bp)	1562
Median transcript length (bp)	438
Total transcript length (bp)	254,557,090
N50 (bp)	4964
Number of genes homologous to the *S. scabiei* var. *canis* sequence	109,185
Number of genes annotated	110,033

### Allergen homologs

To explore known allergens as target molecules for diagnosis, we identified allergen homologs from predicted proteins of *S. scabiei var. nyctereutis* and compared them with the characterized groups 1 through 33 of house dust mite allergens obtained from the previously reported RNA-seq analysis data from draft genome analysis (Table 2)^16^. The numbers of allergens inferred from DNA and RNA analyses were in agreement.

**Table 2:** Number of predicted house dust mite allergen homologs identified in the *Sarcoptes scabiei* RNA-seq data.

Allergen group	Biochemical function	No. of homologs (RNA-seq)	Reference^a^ (DNA-seq)
1	Cysteine protease	12	> 10
2, 22	Lipid-binding protein	1	1
3, 6, 9	Serine protease	40	> 19
4	Amylase	2	0
5, 21	Structural protein	0	0
7	Lipopeptide binding protein	2	2
8	Glutathione S-transferase	5	5
10	Tropomyosin	4	2
11	Paramyosin	6	1
12	Possible chitinase	0	0
13	Fatty acid binding protein	0	1
14	Apolipophorin	2	1
15	Chitinase	11	1
16	Gelsolin	1	1
17	Calcium-binding EF-hand protein	0	5
18	Antimicrobial peptide	0	0
19	Arginine kinase	2	2
20	Lipid-binding protein	1	1
23	Chitin binding protein	15	1
24	Ubiquinol-cytochrome c reductase binding protein	0	1
25	Triosephosphate isomerase	1	1
26	Myosin, light chain	4	11
27	Serpin	11	10
28	Heat shock protein	19	8
29	Cyclophilin	0	12
30	Ferritin	2	3
31	Cofilin	1	1
32	Inorganic pyrophosphatase	1	1
33	αTubulin	2	2

^a^Rider Jr et al.^16^.

### Transcriptional ranking

**Figure 2:**
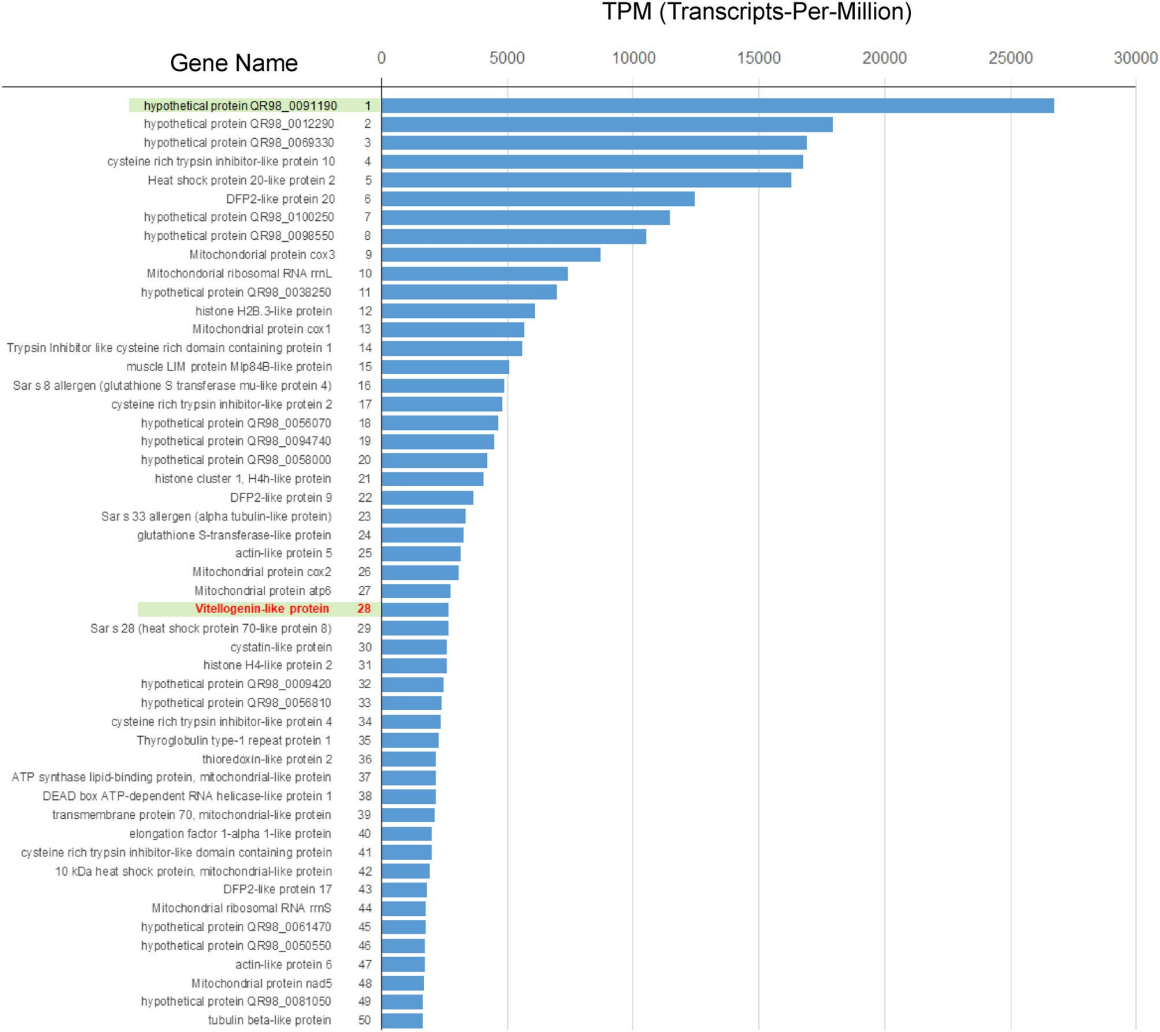
Transcript abundances of annotated genes in the *S. scabies* transcriptome. Top 50 genes ranked according to expression level in terms of transcripts-per-million (TPM) for each sample are shown as a barplot.

The selection of the most adequate molecules for development of a lateral flow immunoassay was based on gene expression levels. The top 50 highly expressed genes out of 162,918 in terms of transcripts-per-million (TPM) for each sample are depicted in Fig. 2 and Supplementary Table 1. The gene with the highest expression was that encoding the hypothetical protein QR98_0091190 (IACW01014455). This protein was ranked significantly higher than the other 14 hypothetical proteins. Two mitochondrial genes were ranked 9th and 10th. In addition, the ranking of seven genes of mitochondrial origin among those of the 50 genes was also distinctive (represented by the light blue highlight) (Supplementary Table 1).

The hypothetical protein QR98_0091190 could be thought to be a candidate diagnostic target. However, the contig encoding this protein has several simple sequence repeats and may be considered as an artifact of the sequence assembly process. Therefore, we confirmed the sequence of the contig encoding QR98_0091190 by Sanger method, using PCR products obtained from total RNA by RT-PCR. A PCR product with an expected size was identified, and the nucleotide sequence of the PCR product was matched to the assembled contig (Supplementary Figure 1). Therefore, it has been confirmed that the assembled contig was correct.

### Selection of candidate antigens

To select candidate antigens, we compared data obtained in this transcriptome study with GenBank data and public proteomic data available for *S. scabies* var *canis*^10^. We selected three antigens, a hypothetical protein QR98_0091190 (IACW01014455), vitellogenin-like protein (IACW01000323), and SMIPP-Cs (the Scabies Mite Inactivated Cysteine Protease Paralogs) (IACW01021976) (Table 3). The reasons for the selection are as follows:

**Table 3:** Selection of three proteins as potential target antigens for scabies diagnosis.

Candidate antigens	Ranking _(based on gene expression)_	Number of Amino acid	The units of MW	pI	Accession no	Reason for selection
Vitellogenin-like protein	28	1938	220,784	5.5	IACW01000323	High expression of mRNA Abundant protein in aqueous extract Low similarity to HDM protein
Hypothetical protein QR98_0091190	1	314	33,023	6.03	IACW01014455	High transcription No homolog
SMIPP-Cc (Sar s 1 allergen) (cysteine protease-like protein 9)	104	315	36,484	5.79	IACW01021976	Mite Allergen 1

*pI* isoelectric point, *HDM* house dust mites.

The hypothetical protein QR98_0091190 (theoretical molecular weight: 33 kDa) was ranked first in terms of gene expression levels in the newly obtained RNA-seq data (Fig. 2) and was a unique protein non-homologous to proteins from other organisms.

SMIPP-Cs (transcript ranked 104th) (molecular weight: 36.48 kDa) was the closest homolog to the group 1 allergens of house dust mites (HDMs) (Table 2), which are proteolytic papain-like cysteine proteases that promote pathogenicity in asthma and allergy^22^ (Supplementary Figure 2). It is also commonly used for a commercially available HDM allergy test. As it is a homolog of the HDM allergen 1 and easily induces antibody formation in the host, we hypothesized that SMIPP-Cc might also act as a stable protein with similar properties, such as retention in host tissue for a long time and ease of uptake by immune cells. Indeed, studies on human scabies mites have shown that SMIPP-C may interfere in the functions of host proteins present in the epidermis or interact with them^22,23^.

Vitellogenin-like protein (transcript ranked 28th) (molecular weight: 220.78 kDa) shared low identity with *D. pteronyssinus* vitellogenin (52%) and *D. farinae* vitellogenin (51%, our inference; refer to the subsection "Evaluation of antigens by western blotting” in detail.) in the BLAST search. Additionally, high protein expression of dog scabies mites was reported in proteome data^18^, with the highest number of spots (11/97 spots) observed in the 2D electrophoresis of aqueous extracts. Although Sar s 28 (heat shock protein 70-like protein; gene expression ranked 29th) and actin-like protein 6 (gene expression ranked 47th) were also shown to have 5/97 spots and 1/97 spots, respectively, in the proteomic data (Table 4), these two proteins were not selected as antigen candidates because of their high homology to proteins in other ticks (there is an 89.18% identity between Sar s 28 and *D. pteronyssinus* heat shock cognate 71 kDa protein and a 95.39% identity between actin-like protein 6 and *Phynus mexicanus* actin) (Table 4).

**Table 4:** Abundances of annotated genes in the *S. scabies* transcriptome.

Ranking (based on gene expression)	Predicted protein	Homologs (tBLASTn analysis)	Percent Identities (amino acid)	Spot number in 97 spots in the proteome analysis^a^	Solubility in aqueous solution
1	hypothetical protein QR98_0091190	No hit	0	0	Insoluble
28	vitellogenin-like protein	*Der.p* vitellogenin-6-like	51.13%	11 spots	Soluble
29	Sar s 28 (heat shock protein 70-like protein 8)	*Der.p* heat shock cognate 71 kDa protein	89.18%	5 spots	Soluble
47	actin-like protein 6	*Phrynus mexicanus* actin	95.39%	1 spot	Soluble
104	Sar s 1 allergen (cysteine protease-like protein 9)	*Der.p cysteine protease*	38.95%	0	Soluble

^a^From Morgan et al.^18^.

### Evaluation of antigens by western blotting

**Figure 3:**
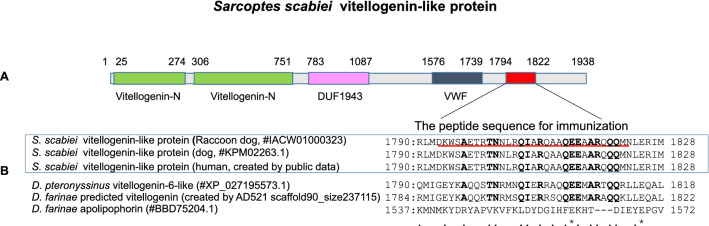
Conserved domain architecture of vitellogenin-like protein in *S. scabiei* and alignment of the sequences of proteins from the three types of scabies mites and two types of house dust mites. (**A**) The vitellogenin N (lipoprotein amino terminal region), DUF1943 (domain of unknown function), and VWD (von Willebrand factor type D domain) domains were predicted based on significant Pfam matches. (**B**) Comparison of sequences proximal to the selected peptide sequence in the vitellogenin-like proteins (red line) from *S. scabiei, D. farinae* (BBD75204.1), and *D. pteronyssinus* (XP_027195573.1). With respect to the amino acid sequence of the peptide used for immunization, the sequence homology was 45%–48% between *S. scabiei* and HDM and 83% between *D. pteronyssinus* and *D. farinae.* Boldfaced text or the use of an asterisk indicates perfect alignment; the dot indicates weak similarity.

**Figure 4:**
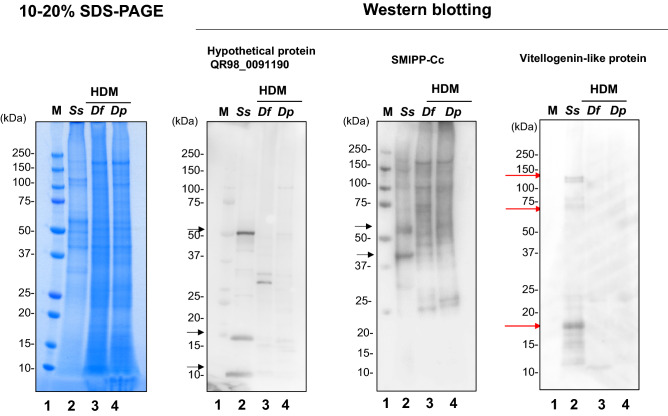
Western blot analysis of the three target antigens for *S. scabies* diagnosis. The whole mite lysates (20 μg/lane) of *S. scabies* and HDMs (*D. farina, D. pteronyssinus*) were immunoblotted with preparations of anti- hypothetical protein QR98_0091190, SMIPP-Cc, and vitellogenin-like protein rabbit polyclonal antibody. Lane 1, molecular weight marker; Lane 2, whole lysate of *S. scabies* (*Ss*); Lane 3, whole lysate of *D. farina* (*Df*); Lane 4, whole lysate of *D. pteronyssinus* (*Dp*); The red arrows indicate the specific bands (17, 70, and 150 kDa) exhibiting reaction to the anti-vitellogenin-like protein antibody. The black arrows indicate the three clear bands (50, 17, and 10 kDa) in the experiment in which the anti-hypothetical protein QR98_0091190 antibody was used and the two bands (40 and 60 kDa) detected using the anti-SMIPP-Cc antibody. These photo data were taken at the same exposure time (4.7 s).

Polyclonal antibodies were generated using the three candidate antigens by immunizing rabbits with the peptides synthesized by selecting amino acid sequences at positions with no or low homology compared to HDM proteins (Fig. 3, Supplementary Figures 2, 3, 4, and Supplementary Table 1). Note that *D. farinae* apolipophorin differed from the other vitellogenins in sequence as shown in the bottom row of Fig. 3. Therefore, we predicted novel vitellogenin-like sequences from scaffolds *of the D. farinae* whole genome and compared them to those of *S. scabies* (Fig. 3). Western blotting analysis using antibodies against the three antigens is depicted in Fig. 4 and Supplementary Figure 6. When anti-vitellogenin-like protein antibody was used, a specific and clear band at 17 kDa and a weak band at 70 kDa and 150 kDa were detected. There was no or low cross-reactivity with HDM proteins. In the case of the anti-hypothetical protein QR98_0091190 antibody, three clear bands were identified at 50, 17, and 10 kDa; a faint band at 30 kDa was also detected in *D. farina*, which indicated that this antibody weakly cross-reacted with HDM. Furthermore, the hypothetical protein QR98_0091190 antigen was found to be soluble in extraction solution containing sodium dodecyl sulfate (SDS) detergent but insoluble in physiological buffer without SDS (Supplementary Figure 5). Therefore, the solubility of this protein in aqueous solutions was low, making it unsuitable for use as a diagnostic antigen.

When anti-SMIPP-Cc antibody was used, strong signals were detected at 40 kDa and 60 kDa. Several bands were also observed for HDMs, confirming the cross-reactivity of this antibody (Fig. 4).

Vitellogenin-like protein was selected based on its high expression level in mites, low identity to HDM proteins, and its solubility, which makes easier the purification process.

### Structural analysis of *S. scabies* vitellogenin-like protein

Domain architecture analysis using Pfam revealed that the amino acid sequence of the *S. scabies* vitellogenin-like protein contains three conserved domains (Fig. 3a): the vitellogenin N domain, the DUF1943 domain, and the von Willebrand factor type D domain. A signal peptide with 18 amino acids (MKTFAGLLLSALIVSASA) was detected in the analysis of the predicted amino acid sequence. In addition, the deduced sequence of vitellogenin-like proteins contained eight N-glycosylation sites (NXS/T) and three ([R/K]–X*n*–[R/K]↓) propeptide cleavage sites (850, 877, and 1886 in the open reading frame (ORF) encoding 1938 aa residues), as predicted by the ProP 1.0 server.

### A molecular phylogenetic tree of arthropod vitellogenin

To investigate the specificity of vitellogenin as a protein molecule and its evolutionary position as a diagnostic antigen, we performed a phylogenetic analysis of vitellogenin in scabies mites and other related organisms.

With respect to raccoon dog mites, one amino acid substitution (P706T) was detected upon comparison to homologs of vitellogenin-like proteins from dog and human mites (Supplementary Figure 4, Supplementary Table 2), and the epitope sequences among the three mite isolates were 100% identical (Fig. 3).

**Figure 5:**
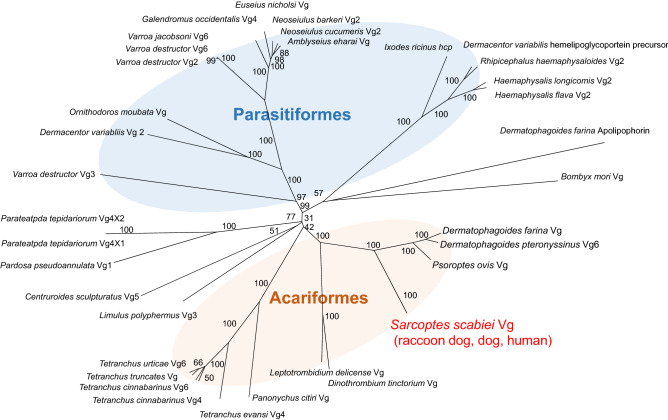
A molecular phylogenetic tree of arthropod vitellogenin was constructed. The DDBJ/GenBank/EMBL accession numbers are follows: IACW01000323 (*Sarcoptes scabiei* var *nyctereutis* Vg like protein), KPM02263.1 (*Sarcoptes scabiei* var *canis* Vg like protein), Creation by scaffold41081_cov61 and 6362_cov109 (*Sarcoptes scabiei* var *hominis* Vg like protein), XP_027195573.1 (*Dermatophagoides pteronyssinus* Vg6), Creation by scaffold NBAF01001615.1 (*Dermatophagoides farina* Vg), Creation by scaffold PQWQ01000014.1 (*Psoroptes ovis* Vg), RWS06019.1 (*Dinothrombium tinctorium* Vg), RWS27858.1 (*Leptotrombidium delicense* Vg), AHN48900.1 (*Panonychus citiri* Vg), AYV89292.1 (*Tetranchus evansi* Vg4), AMO02571.1(*Tetranchus cinnabarinus* Vg4), AMO02572.1 (*Tetranchus cinnabarinus* Vg6), AYV88983.1 (*Tetranchus truncates* Vg), XP015793836.1 (*Tetranchus urticae* Vg6), XP022244466.1 (*Limulus polyphermus* Vg3), XP023225368.1 (*Centruroides sculpturatus* Vg5), AXN69712.1 (*Pardosa pseudoannulata* Vg1), XP015930209.1 (*Parateatpda tepidariorum* Vg4X1), XP015930210.1(*Parateatpda tepidariorum* Vg4X2), XP022657753.1 (*Varroa destructor* Vg3), ABW82681.2 (*Dermacentor variabliis* Vg 2), BAH02666.2 (*Ornithodoros moubata* Vg), AFN88464.1 (*Varroa destructor* Vg2), XP022660223.1 (*Varroa destructor* Vg6), XP022701897.1 (*Varroa jacobsoni* Vg6), XP018494463.1 (*Galendromus occidentalis* Vg4), QCX36526.1 (*Euseius nicholsi* Vg), ASB34116.1 (*Neoseiulus barkeri* Vg2), AGQ56699.1 (*Neoseiulus cucumeris* Vg2), QBZ96191.1 (*Amblyseius eharai* Vg), AJR36491.1 (*Ixodes ricinus* hemolipoglyco-carrier protein CP3), ABD83654.1 (*Dermacentor variabilis* hemelipoglycoportein precursor), QEL09188.1 (*Rhipicephalus haemaphysaloides* Vg2), BAG12081.1 (*Haemaphysalis longicomis* Vg2), AXP34688.1 (*Haemaphysalis flava* Vg2), BBD75204.1 (*Dermatophagoides farina* apolipophorin), and BAA06397.1 (*Bombyx mori* Vg).

Phylogenetic analysis revealed that scabies mite vitellogenin is evolutionarily close to vitellogenin from two HDM species and sheep mite; however, the epitope sequence itself is unique to Sarcoptes, confirming that vitellogenin from scabies mite serves as a strong candidate as a diagnostic antigen for scabies (Fig. 5).

### Lateral flow immunoassay

**Figure 6:**
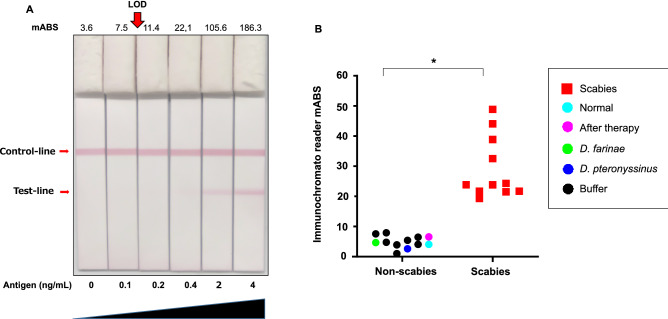
Lateral flow immunoassay for scabies vitellogenin-like protein. (**A**) Sensitivity of immunochromatography test, determined using standard vitellogenin-like protein. A total of 100 μl of serial dilutions (4, 2, 0.4, 0.2, 0.1, and 0 ng/ml) of the synthesized vitellogenin peptide antigen was dispensed onto the cassette and allowed to migrate. Results were read by eye or using an Immunochromato reader after 15 min. Milli Absorbance (mABS) indicated at the top of the lane. The limit of detection (LOD) is indicated using an arrow (0.1–0.2 ng/mL). (**B**) Evaluation of scabies diagnosis using the rapid lateral immunoassay. A total of 100 μL of lysate from skin scrapings of raccoon dogs or whole mite extracts of HDM was dispensed on to the cassette and allowed to migrate. Results determined using the Immunochromato reader are depicted as a dot-plot. It was possible to distinguish between positive (n = 10) and negative (n = 12) readings at a reader value of approximately 10. *; *P* < 0.05 skin scrapings of raccoon dogs

Based on the data of the solubility of the selected antigen in physiological buffers and the high specificity of the antibody against it, a device for lateral flow immunoassay (LFIA) using anti-vitellogenin polyclonal antibodies was developed (Fig. 6). The limit of detection (LOD) or analytical sensitivity of the LFIA was found to be between 0.1 and 0.2 ng/mL of vitellogenin protein evaluated by visual estimation or using an immunochromato reader after 15 min of migration when the synthesized peptide antigen was used (Fig. 6A). Further, positive results were obtained using ten individual skin extracts from scabies-infected raccoon dogs, with the formation of a clear band at the test line (Supplementary Figure 7). One healthy skin extract and one cured skin extract treated with ivermectin gave negative results. The intensities for the mange groups were significantly higher (*P* < 0.05) than those for the non-mange groups (Fig. 6B). The negative result obtained in the LFIA confirms the efficacy of the treatment in the ivermectin-treated racoon dog.

## Discussion

Traditional tests to diagnose scabies in humans still have less than 50% accuracy^6,7^. To overcome this low sensitivity, we tried to develop an immunodiagnostic test for scabies mites based on biochemical and bioinformatic analysis. For the first time, RNA-seq data were obtained by the sequencing of mite RNA from scabies-infected wild raccoon dogs. The database of de novo RNA-seq of *Sarcoptes scabiei* var. *canis*^24^ remained undisclosed and could not be used as a reference. We performed transcriptomic analysis and selected the candidate antigens for diagnosis based on gene expression levels, homology comparisons, number of allergens, and proteomic analysis using data from public databases.

Eventually, vitellogenin-like protein was selected as the diagnostic antigen as it is a soluble protein found in abundance in mite lysates, has low homology to the proteins in HDMs and other mite species, and its specific antibodies exhibit no or low cross-reactivity with HDM proteins. Moreover, western blot analysis revealed the existence of multiple molecules of vitellogenin in the scabies mite. In insects, vitellogenins are large molecules (~200-kD) synthesized in fat bodies through a process that involves substantial structural modification (e.g., glycosylation, lipidation, phosphorylation, and proteolytic cleavage, etc.) of the nascent protein prior to its secretion and transport to the ovaries^25^. Additionally, vitellogenin in ticks (*Ixodes scapularis, Dermacentor variablis, Haemaphysalis longicornis*) and Acari mites (*Tetranychus urticae*) contained multiple proteolytically cleavable subunits with different molecular weights^26–29^. Therefore, it is assumed that scabies mite vitellogenin also has multiple subunits that can be separated by cleavage with proteases. The specific antibodies developed herein were thought to strongly recognize the soluble form of the small, processed vitellogenin rather than the large precursor protein. This is considered to be advantageous in the lateral flow immunoassay (LFIA), wherein small and abundant antigen–antibody complexes flowed easily and efficiently across the surface of the nitrocellulose membrane. A second advantage of the developed LFIA is that the epitope sequence of vitellogenin in this study was 100% identical in mites that are parasitic to humans, dogs, and raccoon dogs, suggesting that the immunodiagnostics of scabies is feasible, not only in raccoon dogs but equally so in humans and other mammals. Another advantage of the developed LFIA is that the homology of vitellogenin with proteins from Acari mites and ticks related to scabies mites, as indicated in our studies, was 50–60%, which makes scabies diagnosis using our assay highly reliable, because LFIA is based on the use of a polyclonal antibody that does not cross-react with antigens from HDMs, possibly ticks, or other Acari mites. It has previously been reported that vitellogenin has an organism-specific amino acid sequence; for example, the expression of vitellogenin in fish has also been put to practical use as a biomarker to detect known endocrine-disrupting chemicals in contaminated environments^30^.

Thus, the LFIA method presented herein is a straightforward, rapid, sensitive, specific, and cost-effective test for the diagnosis of scabies. LFIA is portable and requires no special equipment, as opposed to PCR or ELISA. Additionally, the total cost of the materials used for the assay is typically a few dollars; hence, it could technically be performed at a cost that is 1/100th to 1/10th the cost of other testing methods, considering the capital expenditure on equipment. In addition, the antibodies produced in this study can be stored stably at −80 °C for several years, and the LFIA components can be refrigerated at 4 °C for 1–2 years.

The prototype LFIA yielded 100% sensitivity and specificity when skin samples from raccoon dogs with confirmed scabies mange were tested. However, we only assessed a small number of samples in this study; thus, the assay developed herein remains a prototype at present.

Future studies should involve the assessment of more samples using appropriate negative controls to evaluate the feasibility of the developed assay as a diagnostic tool for scabies*.* Other candidate scabies mite antigen targets can be selected using the same RNA-seq data and public databases used by us and can be used to develop specific antibodies for more accurate, sensitive, and rapid diagnosis of scabies.

Our results provide new insights into the biology of scabies caused by *Sarcoptes scabiei* and new strategies for its diagnosis.

## Methods

### Animal study

All animal experiments were conducted in compliance with the protocol reviewed by the committee for animal experiments (Permit Number: #RKK1111) at the Kanazawa Zoological Gardens in Yokohama, Japan. The mites and eggs of *S. scabiei* were collected from the crusted skin and hair or from a relatively ordinary area naturally shed by a single infected wild raccoon dog, without causing undue suffering to the animal involved. The raccoon dogs used herein were rescued at the Kanazawa Zoological Gardens because of an *S. scabiei* infestation. They were subcutaneously administered ivermectin (Ivomec injection, Merial Japan, Tokyo, Japan) three times at 400 µg/kg every 2 weeks during the first month, along with oral cephalexin (Larixin, Taisho Toyama Pharmaceutical Co., Ltd., Tokyo, Japan) at 20 mg/kg twice a day for the first week^31^.

### Mite collection

The study was conducted on 1000 whole adult mites and eggs, which were washed to reduce bacterial contamination, by modification of a previously reported method^20^. Briefly, the raccoon dog’s skin and hair were washed at 37 °C for 1 h in washing solution [10% SDS, 0.1 μg/μL papain (Wako Pure Chemical Industries, Osaka, Japan), Pronase/ethylenediaminetetraacetic acid (EDTA) solution (Kyokuto Pharmaceutical Industrial, Tokyo, Japan)^32^ containing 25 μg/mL kanamycin (Nacalai Tesque, Kyoto, Japan) and 35 μg/mL chloramphenicol (Wako Pure Chemical Industries)]. Undissolved mites and eggs were then collected using a filter (EASYstrainer, 50 μm mesh, Greiner Bio-One, Kremsmünster, Austria).

### RNA extraction

Total RNA was extracted from freshly collected mites, which had been first washed with the washing solution (as described above), using TRIzol (Thermo Fisher Scientific, MA) and Direct-zol RNA Miniprep (Zymo Research, CA, USA). The quantity and quality of the extracted RNAs were determined using the Qubit fluorometer 2.0 (Thermo Fisher Scientific). Eight RNA samples with a > 20 ng/µL RNA concentration, > 299 ng total RNA amount, and > 1.8 A260/A280 ratio were used for the transcriptomic analysis.

### Library preparation and next generation sequencing

An mRNA-seq library was prepared in accordance with Illumina’s TruSeq RNA sample preparation protocol to minimize contamination from microbial RNA derived from symbiotic poly (A)-less bacteria. Briefly, mRNA was enriched from total RNA using oligo (dT)-attac hed magnetic beads after pretreatment of the products obtained from DNA digestion by DNase I. The enriched mRNA was broken into short fragments for first-strand cDNA (ss cDNA) synthesis, and, subsequently, second-strand cDNA (dsDNA) was synthesized and purified using the QIAquick PCR Purification Kit (Qiagen, Germany), accompanied by adaptor ligation. Finally, ds cDNA fragments of a suitable size were separated by agarose gel electrophoresis for PCR amplification. The prepared library was sequenced to obtain 100 bp paired-end reads using the HiSeq 2000 system (Illumina, CA).

### RNA-seq da ta processing

Raw read data were processed to remove adapter sequences from the 3′ ends, as well as nucleotides with low quality base calls (Quality Score < 30) from both the 5′ and 3′ ends of reads. The trimmed reads were discarded using Cutadapt version 1.9.1 if they contained more than five ambiguous bases (Ns) or if they were shorter than 20 bases^33^. Quality assessment of the raw and trimmed reads was performed using both Cutadapt and FastQC version 0.11.5^34^. The trimmed paired-end reads were assembled de novo using Trinity version 2.2.0^35^, and ORFs of the assembled transcripts were predicted using TransDecoder version 2.1.0^36^ Functional annotation of the assembled transcripts and predicted proteins was performed using Trinotate version 3.0.1^37^. Furthermore, sequence similarity searches of the predicted coding sequences and peptide sequences were performed against the National Center for Biotechnology Information (NCBI) non-redundant nucleotide (nt) and protein (nr) sequence databases using BLASTn and BLASTp version 2.3.0^38^.

The three peptides for immunization were selected based on a comprehensive analysis of antigenic site prediction using multiple algorithms (antigenicity, hydrophilicity/hydrophobicity, and secondary structure, among others).

### Identification of potential allergens

To determine homology of allergen gene sequences between the presently acquired *RNA-seq data* and the draft genome data of dog mites^16^, including data for three mite species besides the scabies mite, 287 allergen genes from *Dermatophagoides farinae*, *Dermatophagoides pteronyssinus*, and *Euroglyphus mayne* were downloaded from NCBI (http://www.ncbi.nlm.nih.gov/).

### Ranking of gene expression

For mapping short-reads in the RNA-seq data, we downloaded a reference genome and a corresponding annotation file from the NCBI taxonomy database (Taxonomy ID: 52283 and GenBank assembly accession GCA_000828355.1, respectively). Bowtie version 2.3.4^39^ and Tophat 2.1.1^40^ were employed to align each short-read to the reference genome, with default parameters, to produce Binary Alignment Map (BAM) files. BAM files were further processed by Rsubread 1.3^41^ an R package used to extract raw read-counts. Raw read-counts were then transformed into the TPM format^42^.

### Preparation of the three peptide antigens and production of antibodies

Three synthetic peptides—TKNYSIKNKRRRKN, aa 300–313 of hypothetical protein QR98_0091190 (314 aa, gene expression ranked 1st) (Supplementary Figure 2); ETTPSDAEENARLS, aa 4–17 at SMIPP-Cc (347 aa, gene expression ranked 104th) (Supplementary Figure 3)^22^; KWSAETRTNNLRQIARQAAQEEAARQQQM, aa 1794–1822 of vitellogenin-like protein (1938 aa, gene expression ranked 28th) —were prepared.

Antibody preparation focused on the three peptides that were linked with a carrier protein (keyhole limpet hemocyanin). The resulting conjugate was homogenized using Freund's complete adjuvant for the initial injection (0.3 mg) at day 0 and with Freund's incomplete adjuvant for the booster injections (0.2 mg) at days 28, 35, and 42. The conjugate was then injected into the rabbits (Japanese white). On day 49, blood was drawn from the immunized rabbits and the antibodies were then harvested.

### Western blotting

The specificity of the three rabbit polyclonal antibodies was analyzed using western blotting. Scabies mites, collected using a method previously reported method^20^. *Dermatophagoides farina* mites (*#* bo002), and *Dermatophagoides pteronyssinus* (*#*ybo002) mites (Biostir, Hiroshima, Japan) were lysed using urea buffer (8 M urea, 50 mM Tris–HCl, pH 7.5, 0.15 M NaCl, and 0.1% Triton X-100) and then sonicated on ice for 1 min using a handheld sonicator (TOMY SEIKO, Tokyo, Japan). Whole lysates were mixed with loading buffer containing 1,4-dithiothreitol and heated at 95 °C for 5 min. Subsequently, the samples were subjected to 10–20% gradient SDS-PAGE. The proteins in the gel were transferred onto a polyvinylidene fluoride (PVDF) membrane (BIO-RAD) using a Trans-Blot Turbo Transfer System (BIO-RAD) at a constant current of 1.3 A for 7 min^43^. After blotting, the membrane was blocked using a PVDF Blocking Reagent for Can Get Signal (TOYOBO, Tokyo, Japan) for 1 h and then treated with the primary rabbit antibodies diluted using Can Get Signal 1 (TOYOBO) (1:2000) at room temperature (20–25 °C) for 16 h; subsequently, it was treated with a horseradish peroxidase-conjugated goat anti-rabbit secondary antibody (#7474; Cell Signaling Technology, USA) in Tris-buffered saline supplemented with 0.1% Tween 20 at room temperature (20–25 °C) for 1 h. Antibody binding was detected using the enhanced chemiluminescence detection method with the SuperSignal West Femto PLUS Chemiluminescent Substrate (Thermo Fisher Scientific) and ImageQuant LAS500 analyzer (Cytiva, MA) or the colorimetric detection method using the 3,3′,5,5′-tetramethylbenzidine substrate AE-1490 EzWestBlue (Atto Corp, Japan).

### Lateral flow immunoassay evaluation

An LFIA cassette for detecting the vitellogenin-like protein of scabies was manufactured by Kyokuto Pharmaceutical Industrial Co. Ltd (Takahagi, Japan) using a rabbit anti-raccoon dog mite vitellogenin like protein polyclonal antibody. In the LFIA, polyclonal antibodies can detect antigens using a capture-and-detect antibody sandwich in the presence of multiple amino acid sequences in the synthetic peptide (30 mer) that the antibody recognizes. A rabbit polyclonal antibody was conjugated to colloidal gold. Briefly, for conjugation, 70 μg of rabbit polyclonal antibody dissolved in PBS (0.7 mg/mL) was added to 1 mL of colloidal gold solution. The mixture was stirred for 16 h at room temperature (20–25 °C), and then 10% (w/v) bovine serum albumin (BSA) solution was added. The labelled bioconjugates were centrifuged at 9,800* g* and 4 °C for 15 min. The obtained precipitate was resuspended with 5 mM Tris–HCl (pH8.2) buffer, containing 150 mM NaCl, BSA (1%, w/v), and sucrose (5%, w/v). The final product was stored at 4 °C in the dark for further use. The detection zone contained immobilized goat anti-rabbit antibodies as a control line and non-labeled anti-vitellogenin-like protein rabbit polyclonal antibody as a test line on nitrocellulose membranes. A visible control line indicated that the gold-labeled antibody flowed along the test strip and performed appropriately.

We studied both relatively ordinary/common mange and crusted mange.

For common mange, 5 mm of the skin containing hair was collected from the carcass of raccoon dogs immediately after their death. For crusted mange, a crusted hairless skin sample was manually collected from the raccoon dog’s body without inflicting pain. The sample was suspended in 500 μL of extraction buffer (lysis step, SDS solution) for a few seconds. Subsequently, the extract was diluted 100 times using the assay solution (50 mM Tris–HCl (pH 7.6), 150 mM NaCl, and 0.05% Tween 20), following which 100 μL was dispensed onto the cassette. The results were visually evaluated or determined using the Immunochromato reader C10066-10 (Hamamatsu photonics, Hamamatsu, Japan) and after 15 min of migration by monitoring the appearance of a red band specific to vitellogenin, along with a band corresponding to the internal control. Pictures of the Immunochromato results were taken using TCR-500 Immuno reader (Trust-Medical, Kasai, Japan) and digital camera. The Tukey test was performed to evaluate statistical significance of results.

### Determination of the analytical sensitivity of the LFIA

Serial dilutions (4, 2, 0.4, 0.2, 0.1, and 0 ng/ml) of the vitellogenin-like derived synthesized peptide (amino acids KWSAETRTNNLRQIARQAAQEEAARQQQM) were prepared using the extraction buffer. Subsequently, 100 μl of each diluted solution was dispensed onto the cassette and allowed to migrate for 15 min. Results were visually evaluated using the Immunochromato reader.

### Sequence comparisons and phylogenetic analysis of vitellogenin

The putative amino acid sequences of the vitellogenin-like protein of *S. scabiei* and other arthropods were obtained from GenBank and aligned using ClustalX version 2.0^44^. The sequence similarities were analyzed using the online BLASTP program available on the NCBI website (https://blast.ncbi.nlm.nih.gov/). The molecular weights and isoelectric points (pIs) of the deduced protein based on the sequences obtained were predicted using the ExPASy proteomics server (http://www.expasy.org). The domain architecture and conserved domains were analyzed using the online servers of Pfam (http://pfam.xfam.org/). The signal peptide was predicted using the SignalP 4.1 server (http://www.cbs.dtu.dk/services/SignalP). The potential O-linked glycosylation sites were predicted using the GPP Prediction Server (http://comp.chem.nottingham.ac.uk/glyco/). The proprotein convertase cleavage sites were predicted using the ProP 1.0 Server (http://www.cbs.dtu.dk/services/ProP/).

Phylogenetic and molecular evolutionary analyses were conducted using MEGA X^45^. A phylogenetic tree was constructed using the neighbor-joining method^46^. The evolutionary distances were computed using the Poisson correction method^47^ and are represented as the number of amino acid substitutions per site. All ambiguous positions were removed for each sequence pair (pairwise deletion option). The statistical confidence of a particular cluster of sequences was evaluated using the bootstrap test with 500 replications^48^. Furthermore, since the amino acid sequence of *D. farina* apolipophorin (BBD75204.1) was found to be significantly less homologous than the vitellogenin amino acid sequence, a new homolog was predicted from the *D. pteronyssinus* draft genome data^49^. Moreover, a vitellogenin homolog of the closely related mite *Psoroptes ovis* was inferred from recent whole-genome data^50^.

### Ethics declaration

This research adheres to the “Japanese Association of Zoos and Aquariums Ethics and Welfare Guidelines” and “Caring for Wildlife: The World Zoo and Aquarium Animal Welfare Strategy”, and was approved by the Kanazawa Zoological Gardens ethical and welfare assessors, Permit Number: #RKK1111 Kanazawa Zoological Gardens, February 20, 2015. All the animal experiments were conducted in compliance with the ARRIVE guidelines. *S. scabiei* mites were collected from the crusted skin naturally shed by a single infected wild raccoon dog without sufferring of the animal involved.

## Supplementary Information

Supplementary Figues and Tables.

Supplementary Information 2.
