# Supplementary material for: Development of a rapid scabies immunodiagnostic assay based on transcriptomic analysis of Sarcoptes scabiei var. nyctereutis
Source: Sci Rep. 2021 Mar 19;11:6455. doi: 10.1038/s41598-021-85290-7 (PMC7979781; doi:10.1038/s41598-021-85290-7)
Supplement: Supplementary file 1 — Supplementary Figues and Tables. [file 41598_2021_85290_MOESM1_ESM.pptx]

## Slide 1
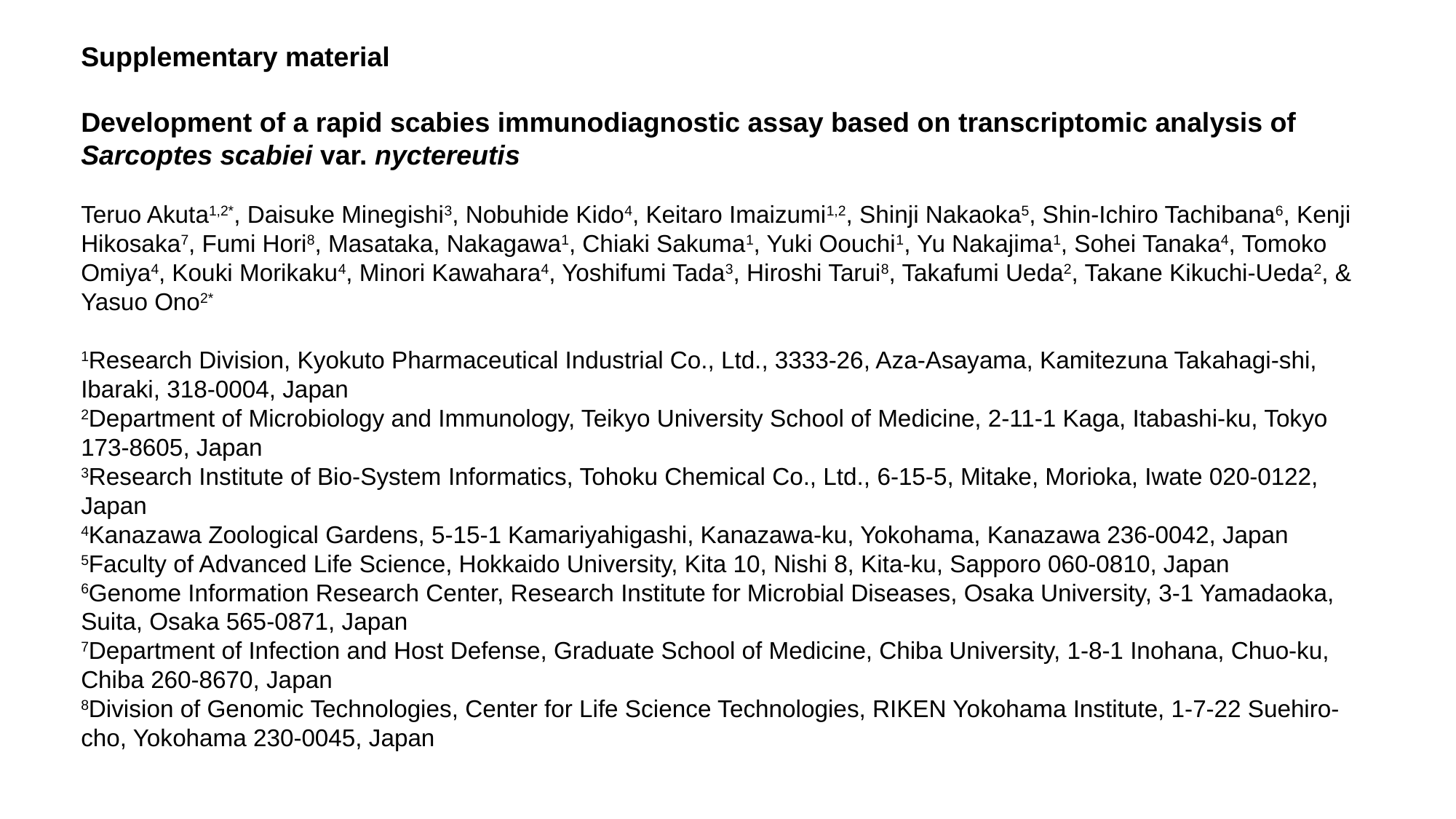

Supplementary material
Development of a rapid scabies immunodiagnostic assay based on transcriptomic analysis of Sarcoptes scabiei var. nyctereutis
Teruo Akuta1,2*, Daisuke Minegishi3, Nobuhide Kido4, Keitaro Imaizumi1,2, Shinji Nakaoka5, Shin-Ichiro Tachibana6, Kenji Hikosaka7, Fumi Hori8, Masataka, Nakagawa1, Chiaki Sakuma1, Yuki Oouchi1, Yu Nakajima1, Sohei Tanaka4, Tomoko Omiya4, Kouki Morikaku4, Minori Kawahara4, Yoshifumi Tada3, Hiroshi Tarui8, Takafumi Ueda2, Takane Kikuchi-Ueda2, & Yasuo Ono2*
1Research Division, Kyokuto Pharmaceutical Industrial Co., Ltd., 3333-26, Aza-Asayama, Kamitezuna Takahagi-shi, Ibaraki, 318-0004, Japan
2Department of Microbiology and Immunology, Teikyo University School of Medicine, 2-11-1 Kaga, Itabashi-ku, Tokyo 173-8605, Japan
3Research Institute of Bio-System Informatics, Tohoku Chemical Co., Ltd., 6-15-5, Mitake, Morioka, Iwate 020-0122, Japan
4Kanazawa Zoological Gardens, 5-15-1 Kamariyahigashi, Kanazawa-ku, Yokohama, Kanazawa 236-0042, Japan
5Faculty of Advanced Life Science, Hokkaido University, Kita 10, Nishi 8, Kita-ku, Sapporo 060-0810, Japan
6Genome Information Research Center, Research Institute for Microbial Diseases, Osaka University, 3-1 Yamadaoka, Suita, Osaka 565-0871, Japan
7Department of Infection and Host Defense, Graduate School of Medicine, Chiba University, 1-8-1 Inohana, Chuo-ku, Chiba 260-8670, Japan
8Division of Genomic Technologies, Center for Life Science Technologies, RIKEN Yokohama Institute, 1-7-22 Suehiro-cho, Yokohama 230-0045, Japan

## Slide 2
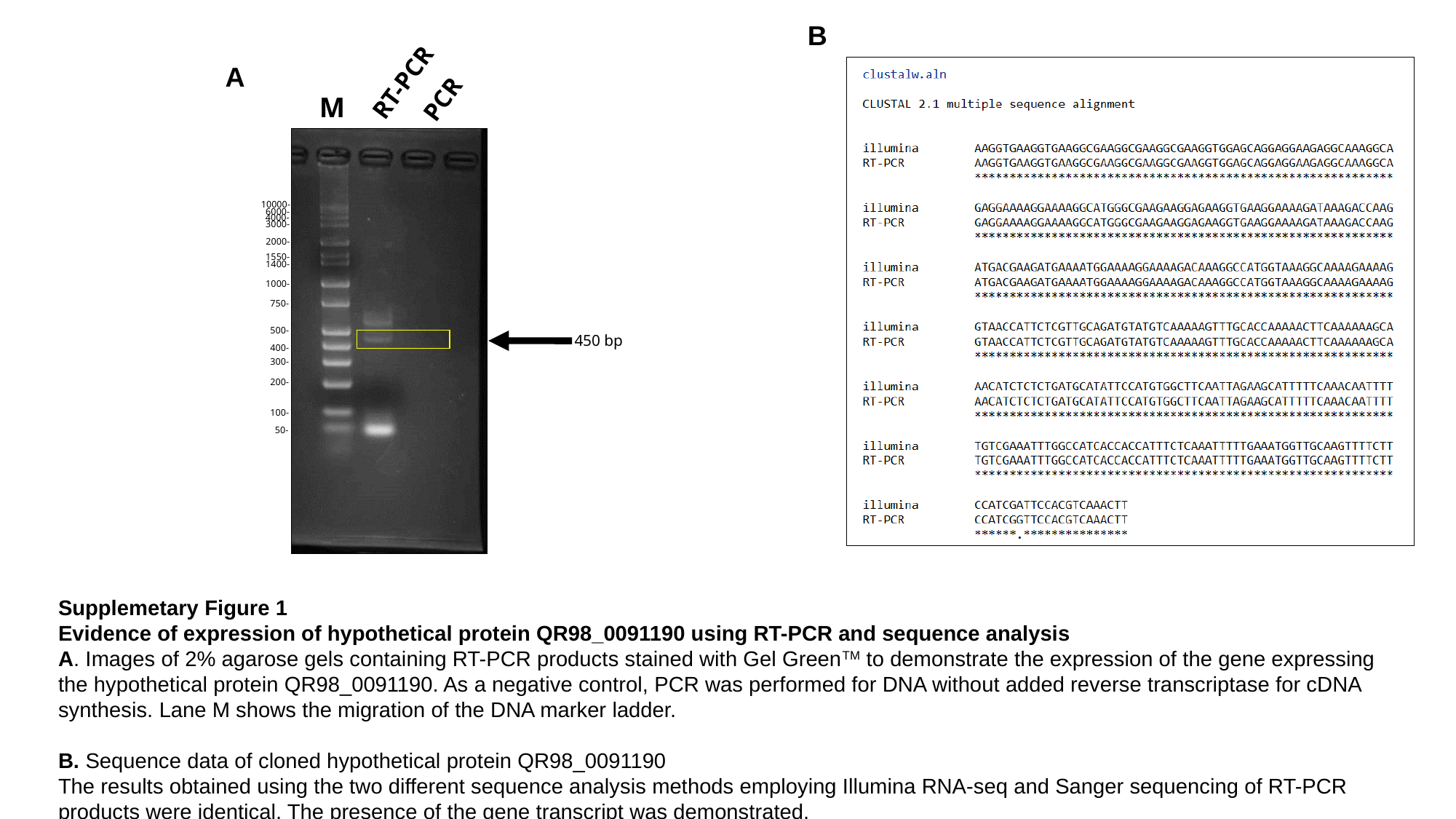

B
A
 RT-PCR
 PCR
M
10000-
6000-
4000-
3000-
2000-
1550-
1400-
1000-
750-
500-
450 bp
400-
300-
200-
100-
50-
Supplemetary Figure 1
Evidence of expression of hypothetical protein QR98_0091190 using RT-PCR and sequence analysis
A. Images of 2% agarose gels containing RT-PCR products stained with Gel GreenTM to demonstrate the expression of the gene expressing the hypothetical protein QR98_0091190. As a negative control, PCR was performed for DNA without added reverse transcriptase for cDNA synthesis. Lane M shows the migration of the DNA marker ladder.
B. Sequence data of cloned hypothetical protein QR98_0091190
The results obtained using the two different sequence analysis methods employing Illumina RNA-seq and Sanger sequencing of RT-PCR products were identical. The presence of the gene transcript was demonstrated.

## Slide 3
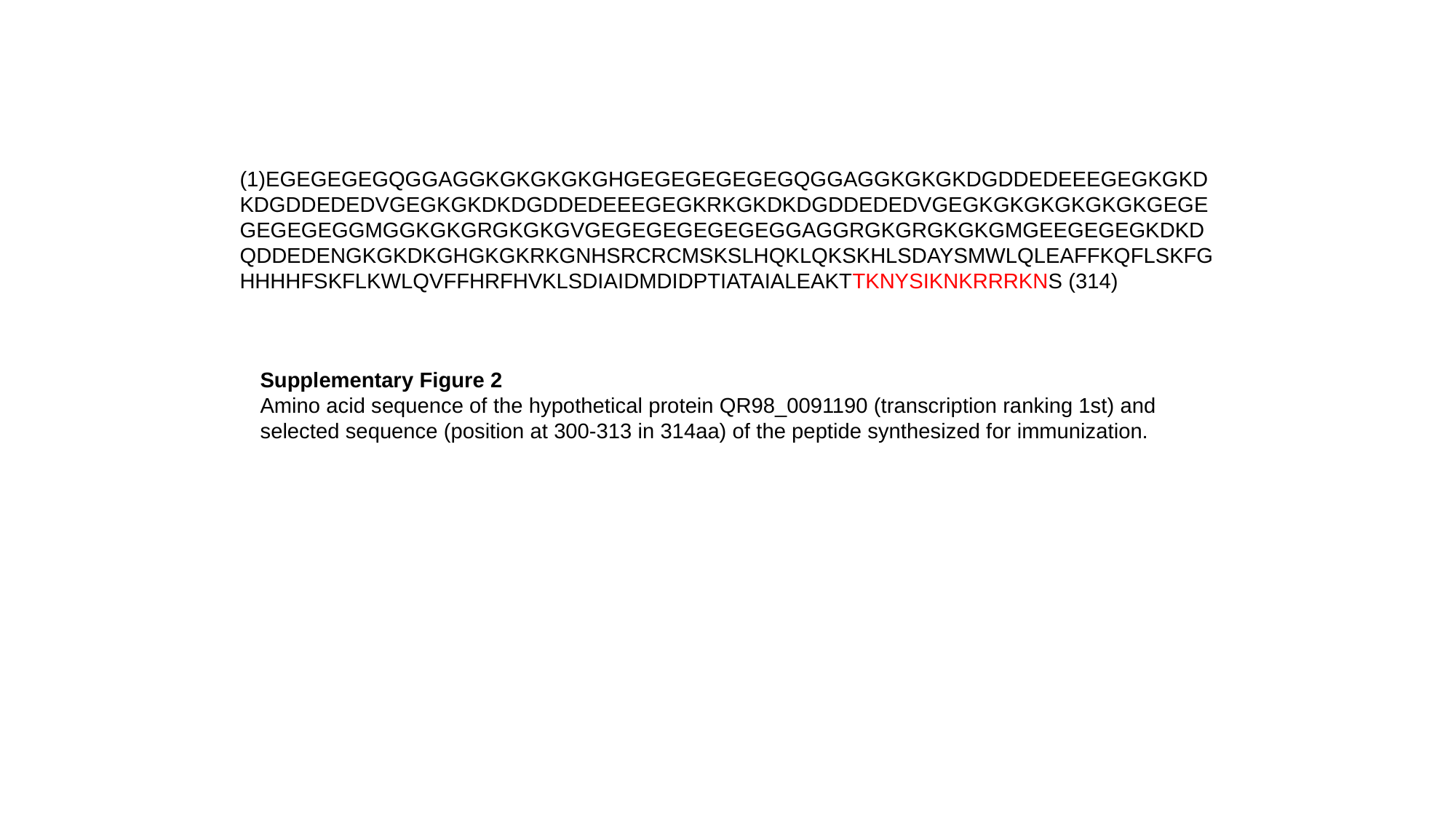

(1)EGEGEGEGQGGAGGKGKGKGKGHGEGEGEGEGEGQGGAGGKGKGKDGDDEDEEEGEGKGKDKDGDDEDEDVGEGKGKDKDGDDEDEEEGEGKRKGKDKDGDDEDEDVGEGKGKGKGKGKGKGEGEGEGEGEGGMGGKGKGRGKGKGVGEGEGEGEGEGEGGAGGRGKGRGKGKGMGEEGEGEGKDKDQDDEDENGKGKDKGHGKGKRKGNHSRCRCMSKSLHQKLQKSKHLSDAYSMWLQLEAFFKQFLSKFGHHHHFSKFLKWLQVFFHRFHVKLSDIAIDMDIDPTIATAIALEAKTTKNYSIKNKRRRKNS (314)
Supplementary Figure 2
Amino acid sequence of the hypothetical protein QR98_0091190 (transcription ranking 1st) and selected sequence (position at 300-313 in 314aa) of the peptide synthesized for immunization.

## Slide 4
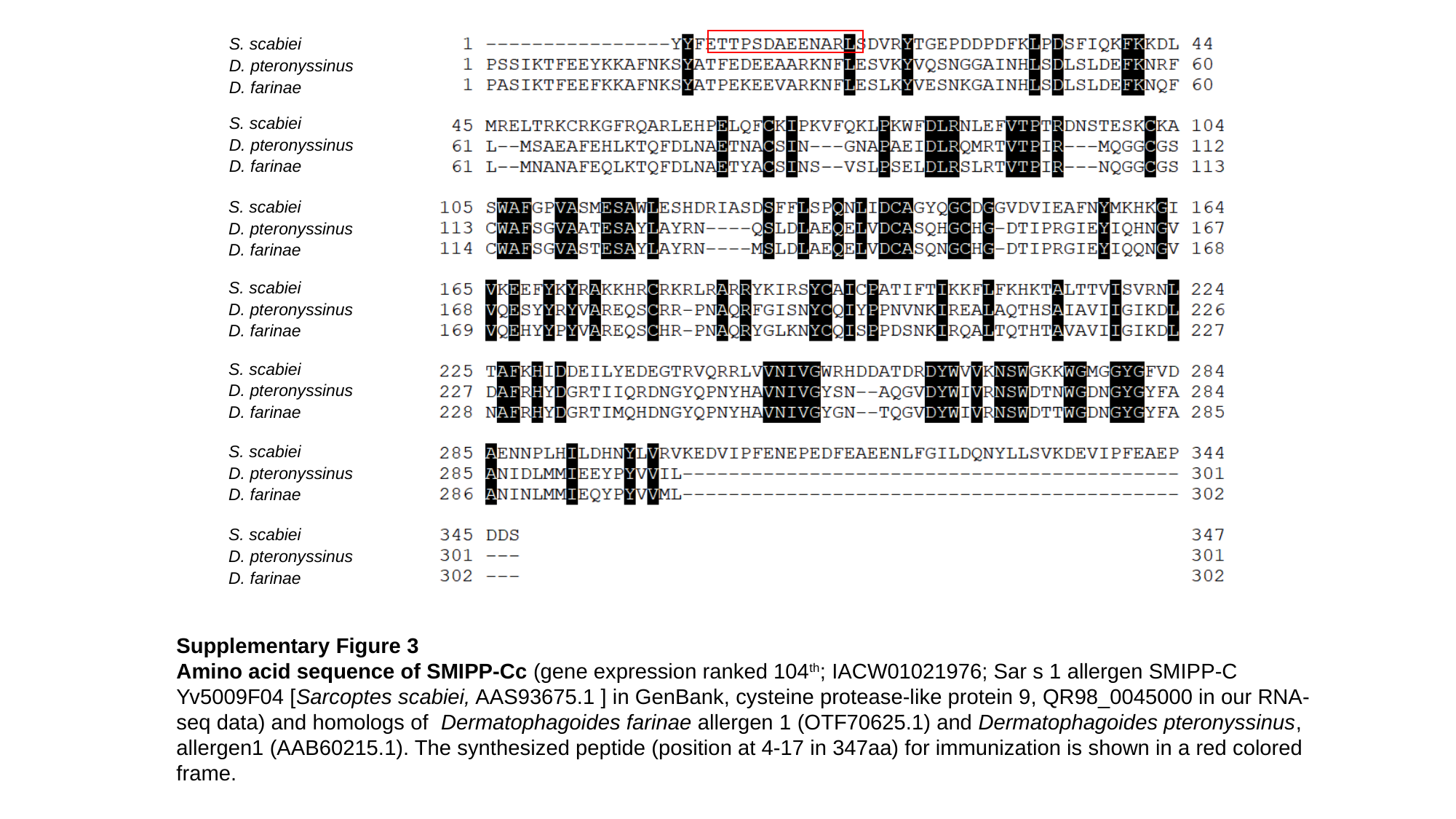

S. scabiei
D. pteronyssinus
D. farinae
S. scabiei
D. pteronyssinus
D. farinae
S. scabiei
D. pteronyssinus
D. farinae
S. scabiei
D. pteronyssinus
D. farinae
S. scabiei
D. pteronyssinus
D. farinae
S. scabiei
D. pteronyssinus
D. farinae
S. scabiei
D. pteronyssinus
D. farinae
Supplementary Figure 3
Amino acid sequence of SMIPP-Cc (gene expression ranked 104th; IACW01021976; Sar s 1 allergen SMIPP-C Yv5009F04 [Sarcoptes scabiei, AAS93675.1 ] in GenBank, cysteine protease-like protein 9, QR98_0045000 in our RNA-seq data) and homologs of Dermatophagoides farinae allergen 1 (OTF70625.1) and Dermatophagoides pteronyssinus, allergen1 (AAB60215.1). The synthesized peptide (position at 4-17 in 347aa) for immunization is shown in a red colored frame.

## Slide 5
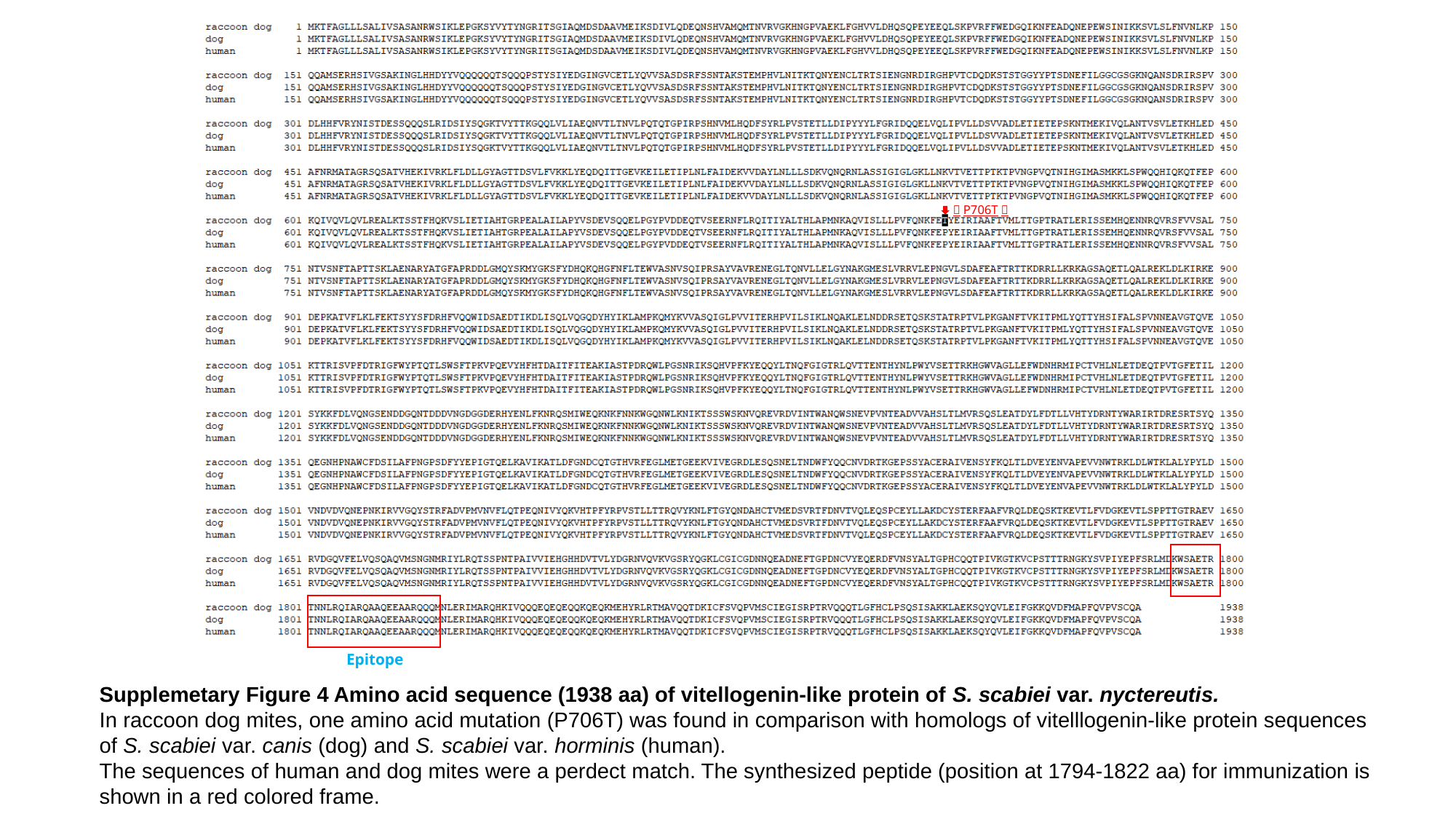

（P706T）
Epitope
Supplemetary Figure 4 Amino acid sequence (1938 aa) of vitellogenin-like protein of S. scabiei var. nyctereutis.
In raccoon dog mites, one amino acid mutation (P706T) was found in comparison with homologs of vitelllogenin-like protein sequences of S. scabiei var. canis (dog) and S. scabiei var. horminis (human).
The sequences of human and dog mites were a perdect match. The synthesized peptide (position at 1794-1822 aa) for immunization is shown in a red colored frame.

## Slide 6
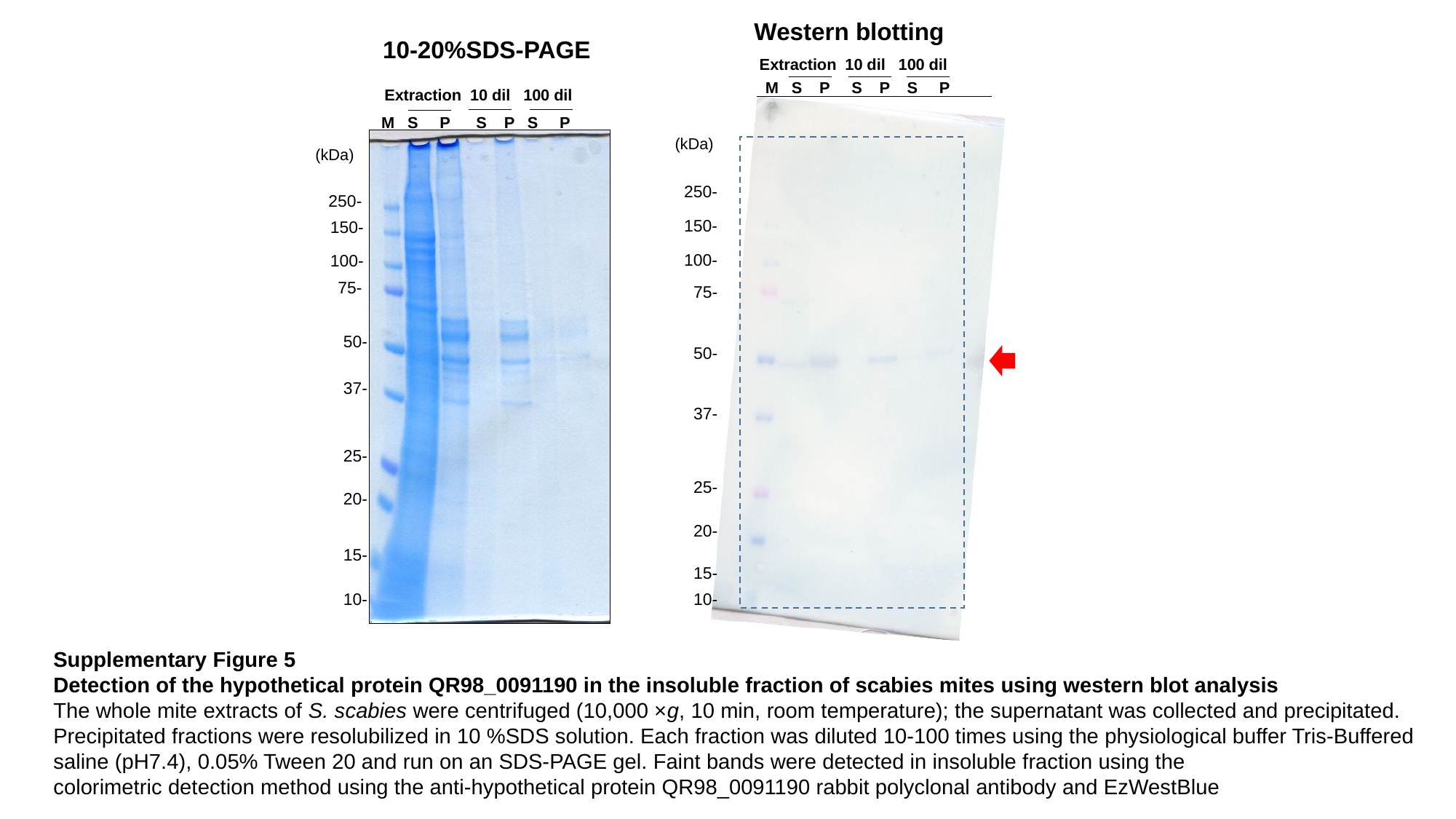

Western blotting
10-20%SDS-PAGE
Extraction 10 dil 100 dil
M S P S P S P
Extraction 10 dil 100 dil
M S P S P S P
(kDa)
(kDa)
250-
250-
150-
150-
100-
100-
75-
75-
50-
50-
37-
37-
25-
25-
20-
20-
15-
15-
10-
10-
Supplementary Figure 5
Detection of the hypothetical protein QR98_0091190 in the insoluble fraction of scabies mites using western blot analysis
The whole mite extracts of S. scabies were centrifuged (10,000 ×g, 10 min, room temperature); the supernatant was collected and precipitated. Precipitated fractions were resolubilized in 10 %SDS solution. Each fraction was diluted 10-100 times using the physiological buffer Tris-Buffered saline (pH7.4), 0.05% Tween 20 and run on an SDS-PAGE gel. Faint bands were detected in insoluble fraction using the
colorimetric detection method using the anti-hypothetical protein QR98_0091190 rabbit polyclonal antibody and EzWestBlue

## Slide 7
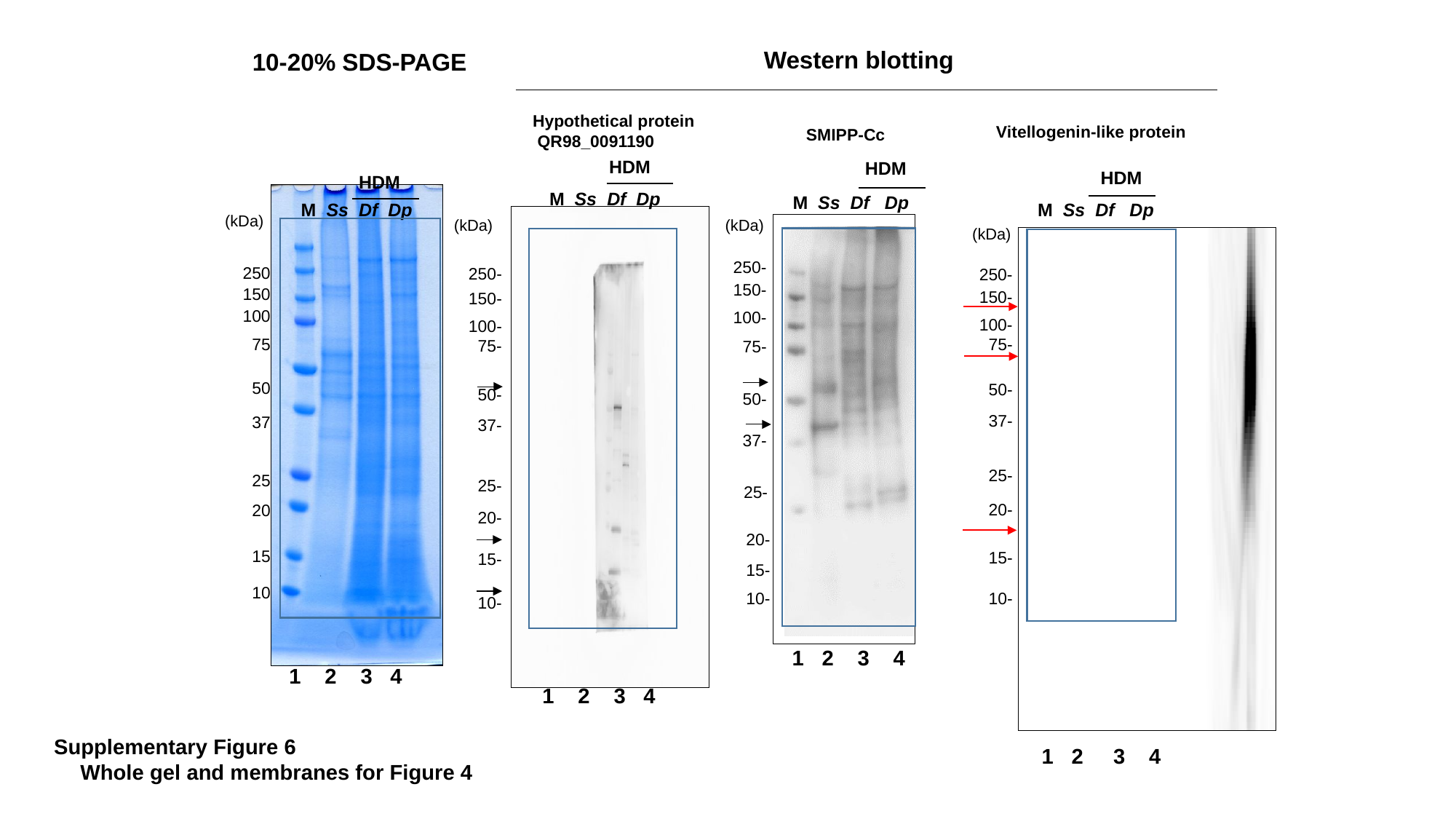

Western blotting
10-20% SDS-PAGE
Hypothetical protein
 QR98_0091190
Vitellogenin-like protein
SMIPP-Cc
HDM
HDM
HDM
HDM
M Ss Df Dp
M Ss Df Dp
M Ss Df Dp
M Ss Df Dp
(kDa)
(kDa)
(kDa)
(kDa)
250-
250-
250-
250-
150-
150-
150-
150-
100-
100-
100-
100-
75-
75-
75-
75-
50-
50-
50-
50-
37-
37-
37-
37-
25-
25-
25-
25-
20-
20-
20-
20-
15-
15-
15-
15-
10-
10-
10-
10-
1 2 3 4
1 2 3 4
1 2 3 4
Supplementary Figure 6
　Whole gel and membranes for Figure 4
1 2 3 4

## Slide 8
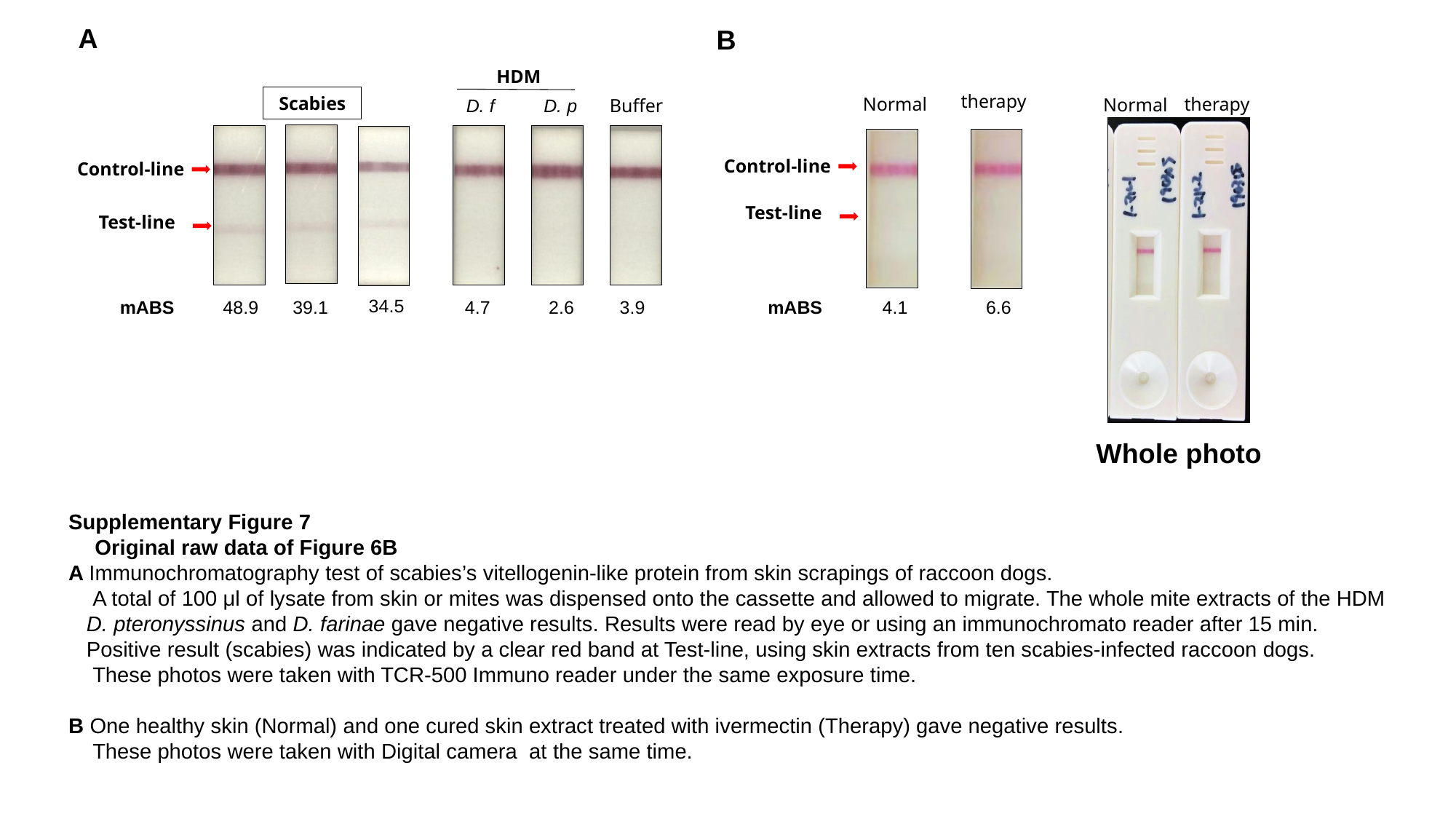

A
B
HDM
 therapy
Scabies
 therapy
Normal
Normal
D. f
D. p
Buffer
Control-line
Control-line
Test-line
Test-line
34.5
mABS
39.1
48.9
4.7
2.6
3.9
mABS
4.1
6.6
Whole photo
Supplementary Figure 7
　Original raw data of Figure 6B
A Immunochromatography test of scabies’s vitellogenin-like protein from skin scrapings of raccoon dogs.
 A total of 100 μl of lysate from skin or mites was dispensed onto the cassette and allowed to migrate. The whole mite extracts of the HDM
 D. pteronyssinus and D. farinae gave negative results. Results were read by eye or using an immunochromato reader after 15 min.
 Positive result (scabies) was indicated by a clear red band at Test-line, using skin extracts from ten scabies-infected raccoon dogs.
 These photos were taken with TCR-500 Immuno reader under the same exposure time.
B One healthy skin (Normal) and one cured skin extract treated with ivermectin (Therapy) gave negative results.
 These photos were taken with Digital camera at the same time.

## Slide 9
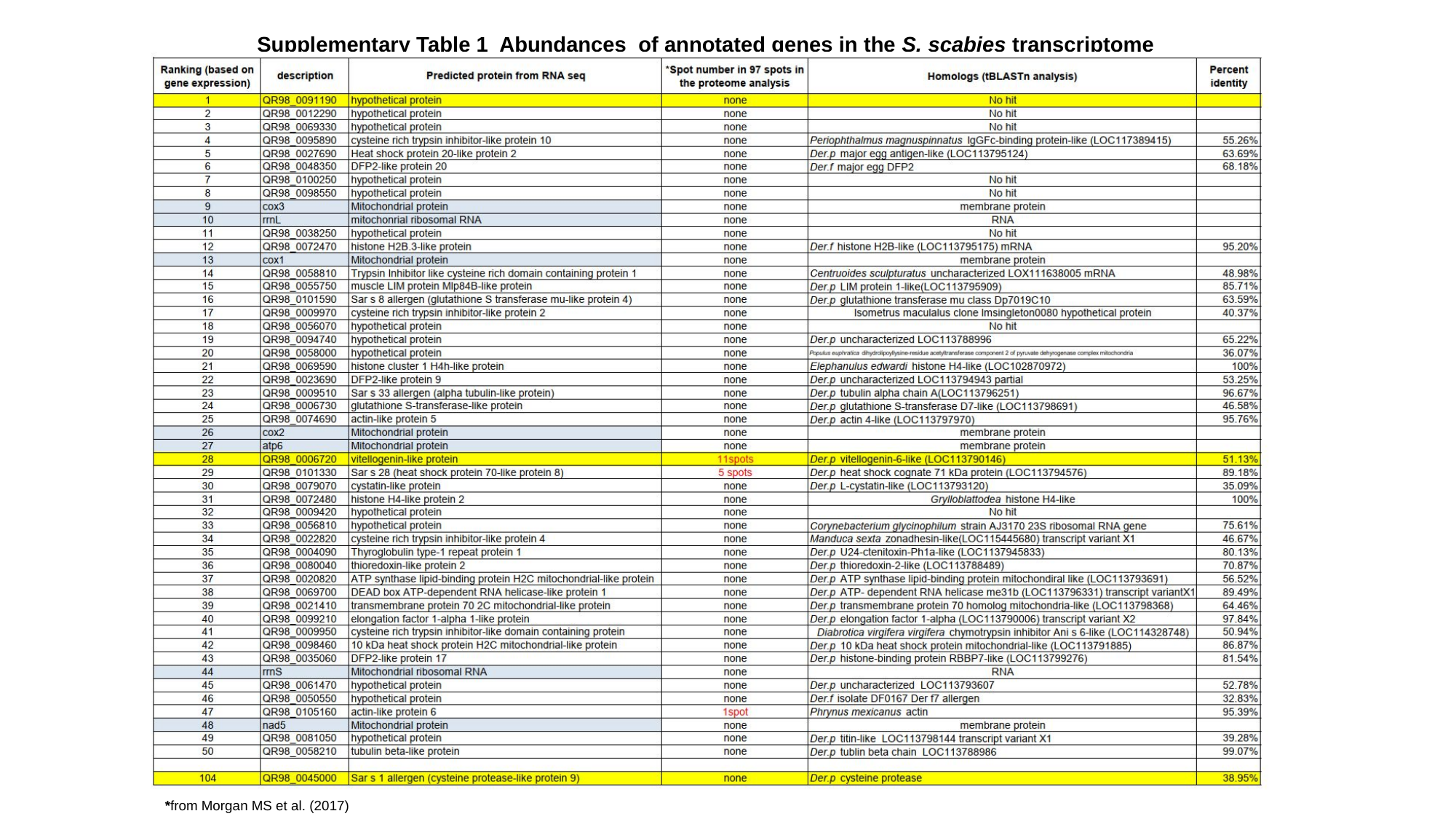

Supplementary Table 1 Abundances  of annotated genes in the S. scabies transcriptome
*from Morgan MS et al. (2017)

## Slide 10
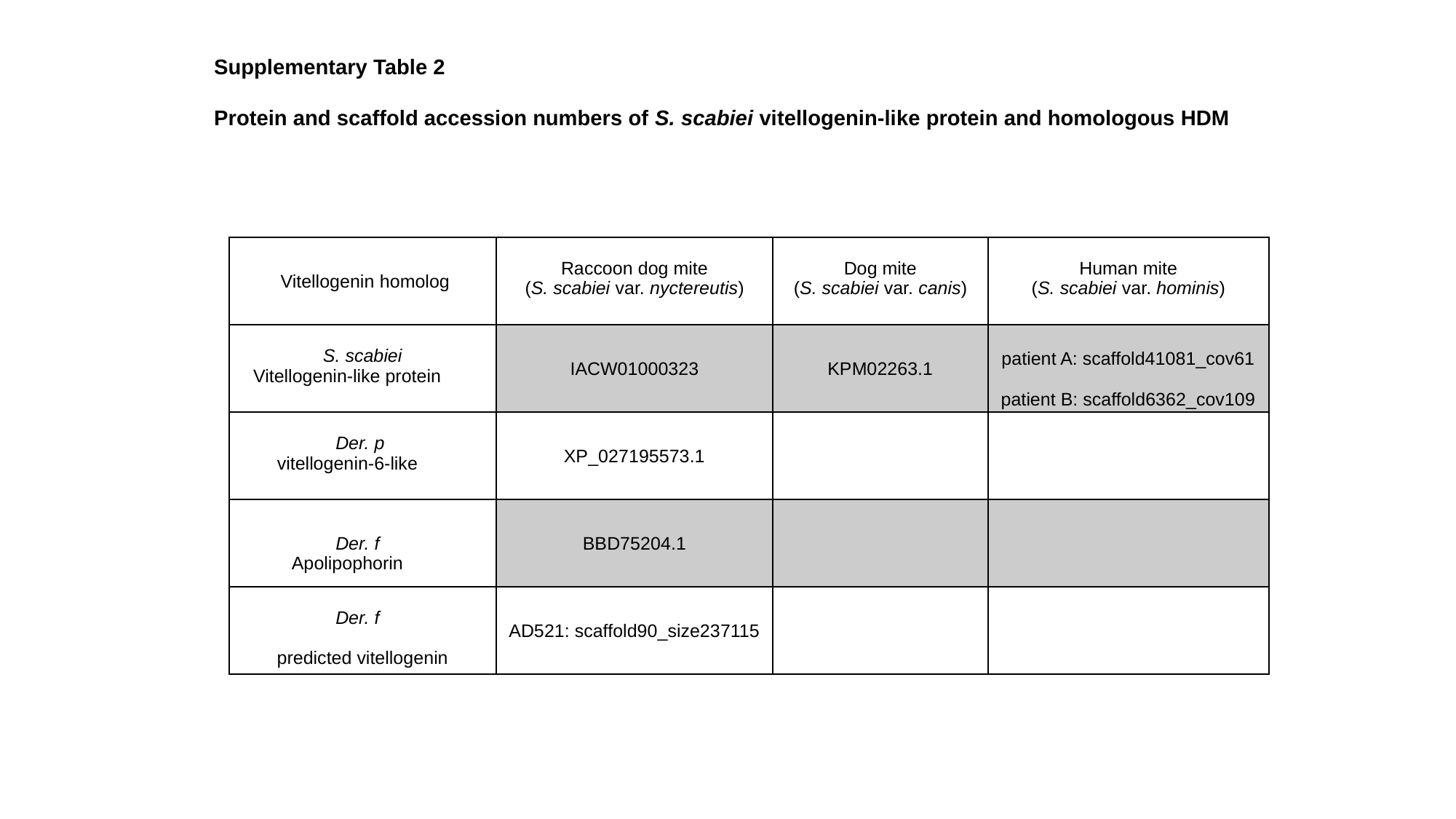

Supplementary Table 2
Protein and scaffold accession numbers of S. scabiei vitellogenin-like protein and homologous HDM
| Vitellogenin homolog | Raccoon dog mite (S. scabiei var. nyctereutis) | Dog mite (S. scabiei var. canis) | Human mite (S. scabiei var. hominis) |
| --- | --- | --- | --- |
| S. scabiei Vitellogenin-like protein | IACW01000323 | KPM02263.1 | patient A: scaffold41081\_cov61 patient B: scaffold6362\_cov109 |
| Der. p vitellogenin-6-like | XP\_027195573.1 | | |
| Der. f Apolipophorin | BBD75204.1 | | |
| Der. f predicted vitellogenin | AD521: scaffold90\_size237115 | | |

## Slide 11
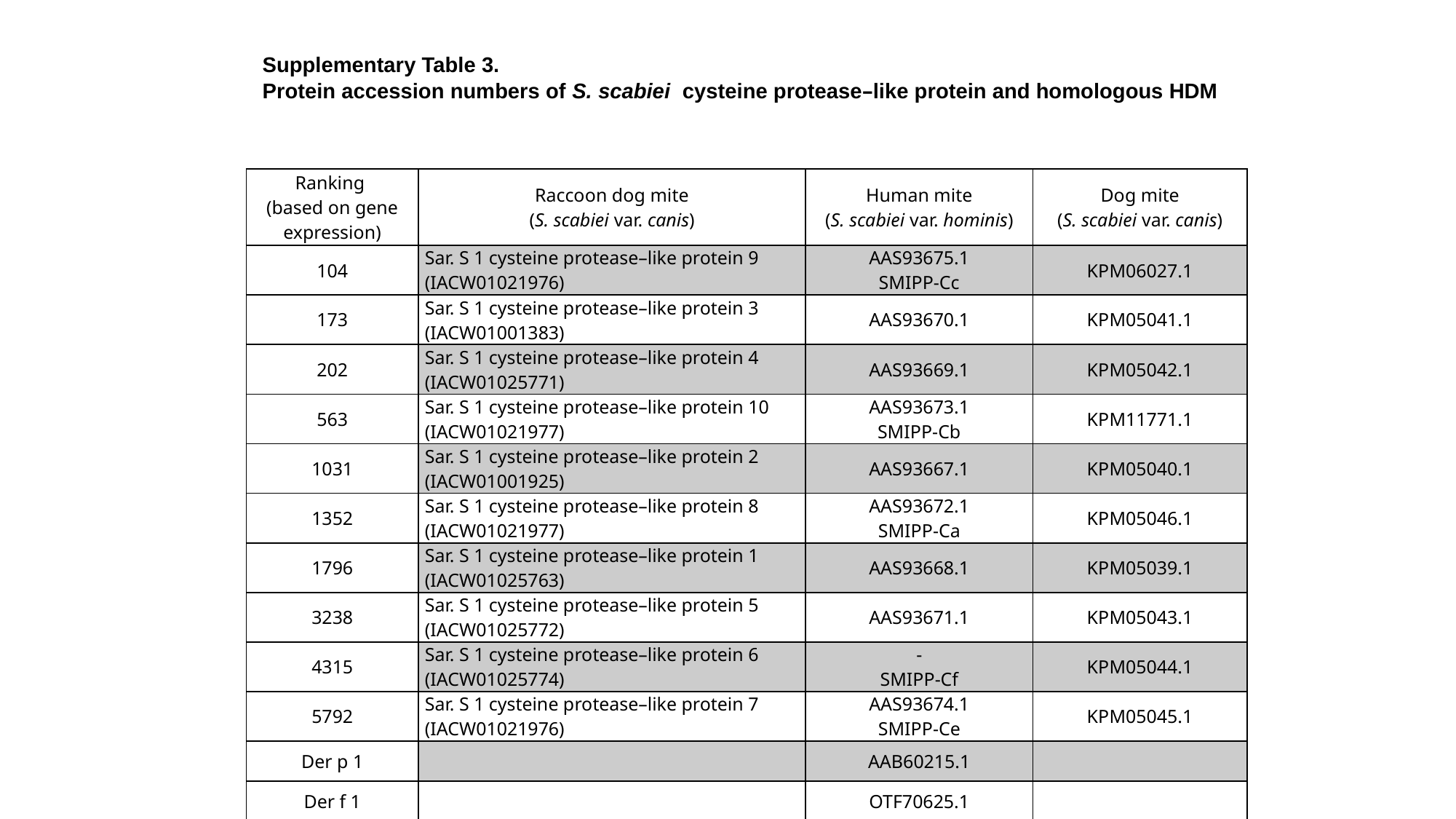

Supplementary Table 3.
Protein accession numbers of S. scabiei cysteine protease–like protein and homologous HDM
| Ranking (based on gene expression) | Raccoon dog mite (S. scabiei var. canis) | Human mite (S. scabiei var. hominis) | Dog mite (S. scabiei var. canis) |
| --- | --- | --- | --- |
| 104 | Sar. S 1 cysteine protease–like protein 9 (IACW01021976) | AAS93675.1 SMIPP-Cc | KPM06027.1 |
| 173 | Sar. S 1 cysteine protease–like protein 3 (IACW01001383) | AAS93670.1 | KPM05041.1 |
| 202 | Sar. S 1 cysteine protease–like protein 4 (IACW01025771) | AAS93669.1 | KPM05042.1 |
| 563 | Sar. S 1 cysteine protease–like protein 10 (IACW01021977) | AAS93673.1 SMIPP-Cb | KPM11771.1 |
| 1031 | Sar. S 1 cysteine protease–like protein 2 (IACW01001925) | AAS93667.1 | KPM05040.1 |
| 1352 | Sar. S 1 cysteine protease–like protein 8 (IACW01021977) | AAS93672.1 SMIPP-Ca | KPM05046.1 |
| 1796 | Sar. S 1 cysteine protease–like protein 1 (IACW01025763) | AAS93668.1 | KPM05039.1 |
| 3238 | Sar. S 1 cysteine protease–like protein 5 (IACW01025772) | AAS93671.1 | KPM05043.1 |
| 4315 | Sar. S 1 cysteine protease–like protein 6 (IACW01025774) | - SMIPP-Cf | KPM05044.1 |
| 5792 | Sar. S 1 cysteine protease–like protein 7 (IACW01021976) | AAS93674.1 SMIPP-Ce | KPM05045.1 |
| Der p 1 | | AAB60215.1 | |
| Der f 1 | | OTF70625.1 | |
