# Supplementary material for: Development of a rapid scabies immunodiagnostic assay based on transcriptomic analysis of Sarcoptes scabiei var. nyctereutis
Source: Sci Rep. 2021 Mar 19;11:6455. doi: 10.1038/s41598-021-85290-7 (PMC7979781; doi:10.1038/s41598-021-85290-7)
Supplement: Supplementary file 2 — Supplementary Information 2. [file 41598_2021_85290_MOESM2_ESM.docx]

**Supplementary material**

**Development of a rapid scabies immunodiagnostic assay based on transcriptomic analysis of *Sarcoptes scabiei* var. *nyctereutis***

Teruo Akuta^1,2*^, Daisuke Minegishi^3^, Nobuhide Kido^4^, Keitaro Imaizumi^1,2^, Shinji Nakaoka^5^, Shin-Ichiro Tachibana^6^, Kenji Hikosaka^7^, Fumi Hori^8^, Masataka, Nakagawa^1^, Chiaki Sakuma^1^, Yuki Oouchi^1^, Yu Nakajima^1^, Sohei Tanaka^4^, Tomoko Omiya^4^, Kouki Morikaku^4^, Minori Kawahara^4^, Yoshifumi Tada^3^, Hiroshi Tarui^8^, Takafumi Ueda^2^, Takane Kikuchi-Ueda^2^, & Yasuo Ono^2*^

^1^ Research Division, Kyokuto Pharmaceutical Industrial Co., Ltd., 3333-26, Aza-Asayama, Kamitezuna Takahagi-shi, Ibaraki, 318-0004, Japan

^2^Department of Microbiology and Immunology, Teikyo University School of Medicine, 2-11-1 Kaga, Itabashi-ku, Tokyo 173-8605, Japan

^3^Research Institute of Bio-System Informatics, Tohoku Chemical Co., Ltd., 6-15-5, Mitake, Morioka, Iwate 020-0122, Japan

^4^Kanazawa Zoological Gardens, 5-15-1 Kamariyahigashi, Kanazawa-ku, Yokohama, Kanazawa 236-0042, Japan

^5^Faculty of Advanced Life Science, Hokkaido University, Kita 10, Nishi 8, Kita-ku, Sapporo 060-0810, Japan

^6^Genome Information Research Center, Research Institute for Microbial Diseases, Osaka University, 3-1 Yamadaoka, Suita, Osaka 565-0871, Japan

^7^Department of Infection and Host Defense, Graduate School of Medicine, Chiba University, 1-8-1 Inohana, Chuo-ku, Chiba 260-8670, Japan

^8^Division of Genomic Technologies, Center for Life Science Technologies, RIKEN Yokohama Institute, 1-7-22 Suehiro-cho, Yokohama 230-0045, Japan

**Material and Method**

**Confirmation of mRNA sequence of Hypothetical protein QR98_0091190**

The mite total RNAs treated by DNase (TaKaRa Bio, Kusatsu, Japan) were used as template for the RT-PCR amplification of the cDNA fragment of the hypothetical protein QR98_0091190, using the primers Forward: 5’-AAGGTGAAGGTGAAGGCGAAGGC-3’ and Reverse: 5’-GCCTCCAAAGCGATAGCGGTAGC-3’ and the SuperScript^®^ III RT/Platinum *Taq* mix (Thermo Fisher Scientific). The first-strand cDNA synthesis of hypothetical protein QR98_0091190 was performed at 55 °C for 30 min. Reverse transcriptase was then inactivated at 94 °C for 2 min. Standard cycling conditions consisted of 40 cycles of denaturation at 94 °C for 15 s, annealing at 55 °C for 30 s, and extension at 68 °C for 1.5 min, followed by a final extension at 68 °C for 5 min. All PCR products were separated on 2 % agarose gels (Nippon Gene, Tokyo, Japan) in 1× Tris-acetate-EDTA buffer and visualized after staining with GelGreen™ dye (Biotium, Fremont, CA). Additionally, KOD plus-ver2 DNA polymerase (Toyobo, Osaka, Japan) was used without reverse transcriptase to prevent the amplification of contaminating genomic DNA. The RT-PCR products were cloned into the pCR2.1 TA cloning vector (Thermo Fisher Scientific) and sequenced by Sanger’s method.
